# Host lung microbiota promotes malaria-associated acute respiratory distress syndrome

**DOI:** 10.1038/s41467-022-31301-8

**Published:** 2022-06-29

**Authors:** Debanjan Mukherjee, Ângelo Ferreira Chora, Jean-Christophe Lone, Ricardo S. Ramiro, Birte Blankenhaus, Karine Serre, Mário Ramirez, Isabel Gordo, Marc Veldhoen, Patrick Varga-Weisz, Maria M. Mota

**Affiliations:** 1 Instituto de Medicina Molecular, Faculdade de Medicina da Universidade de Lisboa, 1649-028 Lisboa, Portugal; 2 School of Life Sciences, University of Essex, Colchester, UK; 3 Instituto Gulbenkian de Ciência, Oeiras, Portugal; 4 São Paulo Excellence Chair, Department of Genetics, Evolution, Microbiology and Immunology, Institute of Biology, University of Campinas, Campinas, Brazil

**Keywords:** Parasite host response, Malaria, Respiratory distress syndrome

## Abstract

Severe malaria can manifest itself with a variety of well-recognized clinical phenotypes that are highly predictive of death – severe anaemia, coma (cerebral malaria), multiple organ failure, and respiratory distress. The reasons why an infected individual develops one pathology rather than another remain poorly understood. Here we use distinct rodent models of infection to show that the host microbiota is a contributing factor for the development of respiratory distress syndrome and host mortality in the context of malaria infections (malaria-associated acute respiratory distress syndrome, MA-ARDS). We show that parasite sequestration in the lung results in sustained immune activation. Subsequent production of the anti-inflammatory cytokine IL-10 by T cells compromises microbial control, leading to severe lung disease. Notably, bacterial clearance with linezolid, an antibiotic commonly used in the clinical setting to control lung-associated bacterial infections, prevents MA-ARDS-associated lethality. Thus, we propose that the host’s anti-inflammatory response to limit tissue damage can result in loss of microbial control, which promotes MA-ARDS. This must be considered when intervening against life-threatening respiratory complications.

## Abstract (web summary)

The reasons why malaria manifests with a variety of well-recognized clinical phenotypes remain poorly understood. Here, using distinct rodent models, the authors reveal that the microbiota colonizing the lung promotes respiratory distress syndrome and mortality during malaria infections.

## Introduction

Acute respiratory distress syndrome (ARDS) consists of a pattern of clinical manifestations of severe hypoxemia and altered respiratory system mechanics due to diffuse alveolar damage. It is present in ~10% of all patients in intensive care units worldwide and mortality remains high at 30–40% in most studies^1^. ARDS has a heterogeneous aetiology, with many possible causes, involving either pulmonary or extra-pulmonary inciting events. However, most cases of ARDS are caused by local (lung) or systemic infections by a variety of pathogens^2–4^.

Malaria remains one of the greatest scourges of humanity—more than 200 million new infections and 400 thousand deaths still occur every year (World Health Organization, 2020)^5^. The reasons why some individuals develop severe malaria and others do not are only partially understood. Both host genetic factors, mostly influencing the characteristics of the red blood cell (RBC)^6,7^, and parasite factors, such as the *var* gene polymorphisms in *P. falciparum*^8,9^, which influence sequestration, i.e., the adherence of infected RBCs (iRBCs) to the vascular endothelium, have been documented as having a critical role in affording protection or being associated with a greater likelihood of developing severe malarial pathology. However, severe malaria can manifest itself in very different ways, with a variety of well-recognized clinical phenotypes, which are highly predictive of death, and that may occur alone or in combination. These include severe anaemia, coma (cerebral malaria), renal failure, and respiratory distress. Even less known are the reasons why an infected individual develops one pathology rather than another, although this does not appear to be due to chance, because these different manifestations have strong associations with factors such as the host’s age and the intensity of malaria transmission in the area where they live^10,11^.

ARDS is unique amongst severe malaria manifestations. It is the most severe form of all respiratory complications associated with malaria, resulting in high mortality rates despite adequate therapeutic management^12,13^. It has been suggested that as many as 20–30% of the patients with severe and complicated malaria requiring admission to intensive care units may develop malaria-associated ARDS (MA-ARDS), which often develops after anti-malarial treatment has been initiated^14^.

The influence of microbiota in homeostasis has recently taken the central stage, as revealed by its critical systemic effects throughout the meta-organism^15^. Indeed, a ‘deviated’ repertoire of the gut microbiome, commonly referred to as ‘intestinal dysbiosis’, has been epidemiologically—and sometimes causally—associated with a wide variety of disorders^16,17^. However, the contribution of the mammalian host microbiota on malaria disease severity has only now started to emerge. Recent studies have demonstrated that *Plasmodium* infections may impact on the composition of commensal bacteria populations. In fact, two independent studies show that infection with two distinct rodents *Plasmodium* spp. (*Plasmodium yoelii* and *Plasmodium berghei*) resulted in alterations in the gut microbiota profile^18,19^. Moreover, it has been proposed that such differences in gut microbiota profiles are associated with distinct clinical outcomes of infection, that is, cerebral and intestinal pathologies^18^. Importantly, microbial colonization occurs on all body surfaces that are exposed to the external environment, including the bronchopulmonary tract. Akin to the association between microbial communities colonizing the gut and specific pathologies^20^, it has been shown that alterations in the composition in this local microbial community are associated with the exacerbation of several pulmonary disorders, including chronic obstructive pulmonary disease (COPD), asthma and cystic fibrosis^21,22^ as well as lung cancer^23^.

Here, we show that alterations of the host microbiota colonizing the lung are a contributing factor of host mortality due to MA-ARDS, a severe malaria pathology. Sequestration of *Plasmodium* parasites causes persistent immune activation, ultimately resulting in the production of the anti-inflammatory cytokine IL-10. IL-10 production by T cells facilitates the outgrowth of the local microbiota, contributing to the establishment of MA-ARDS. Our data further show that antibiotic treatment or blockade of the cellular or molecular immune mediators of microbiota alterations significantly suppress lung-associated pathology, which is sufficient to prevent ARDS.

## Results

### Lung microbiota dysbiosis is associated with ARDS during malaria infections

**Fig. 1:**
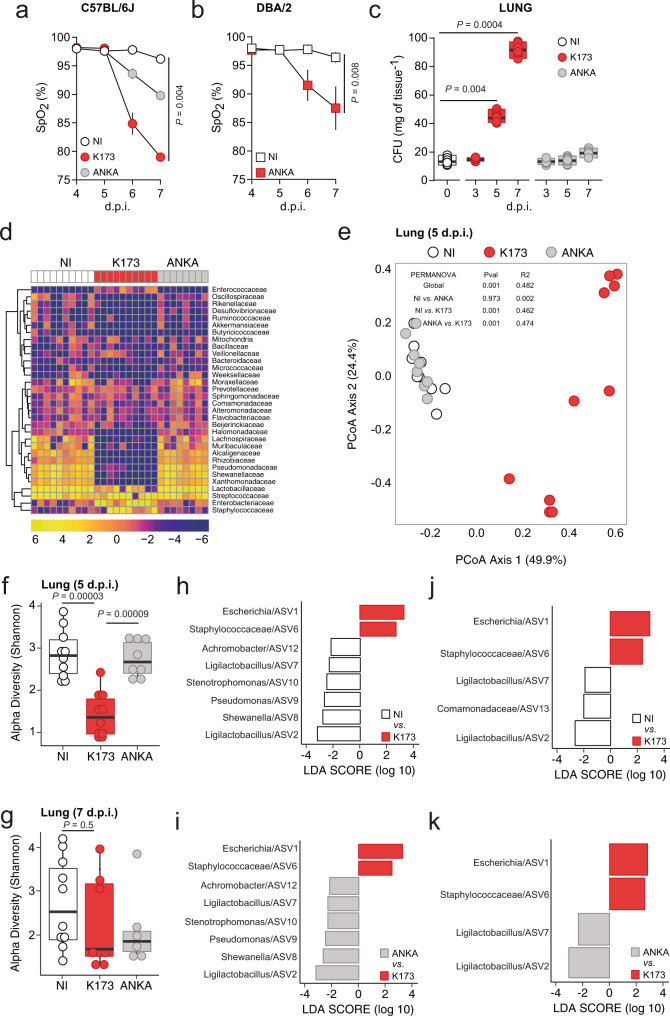
Lung microbiota dysbiosis is associated with ARDS during malaria infections. **a**, **b** Time course measurement of oxygen saturation levels (SpO_2_) as determined by pulse-oximetry (Kruskal–Wallis) of C57BL/6J mice **a** infected with *Pb*K173, *Pb*ANKA or non-infected (NI) controls (*n* = 8 per group; *N* = 2) or DBA/2 mice **b** infected with *Pb*ANKA or non-infected (NI) controls (*n* = 5 per group; *N* = 1). **a**, **b** Data are represented as mean with the bars representing s.e.m**. c** Time course measurement of CFUs (Kruskal–Wallis) in the lungs of C57BL/6J mice infected with *Pb*K173 or *Pb*ANKA at day 3, 5 and 7 post infection (p.i.) and NI (*n* = 8 per group; *N* = 2). Data are represented as floating bar plots (minimum to maximum) with line at the middle representing median. **d** Heatmap of relative abundance of major microbial families in the lungs of C57BL/6J mice 5 days p.i. with *Pb*K173 or *Pb*ANKA and NI (*n* = 8–10 per group, *N* = 1) is shown using a pseudo-logarithmic scale; each column represents one mouse. Each row represents a bacterial family. Rows were clustered using hierarchical clustering with Euclidean distance. **e** Beta diversity of lung microbial communities of C57BL/6J mice 5 days p.i. with *Pb*K173 or *Pb*ANKA and NI (*n* = 8–10, *N* = 1). ASVs space was reduced using principal coordinate analysis (PCoA) with Bray-Curtis distance. The first and second principal coordinates were plotted, as well as the variability explained by each principal coordinates (values in brackets). **f**, **g** Alpha diversity (Shannon) of lung microbial composition upon infection of C57BL/6J mice on (**f**) day 5 p.i with *Pb*K173 or *Pb*ANKA and NI; **g** day 7 p.i with *Pb*K173 or *Pb*ANKA and NI (*n* = 6–10 per group, *N* = 1). Data are represented as boxplots. The lower and upper hinges represent the first and third quartiles. The upper whisker extends to the largest value but no further than 1.5 * inter-quartile range (IQR). The lower whisker extends to the smallest value or most 1.5 * IQR. The Centre hinge is the second quartile. For group comparison, we used Kruskal–Wallis followed by two-sided Mann–Whitney post hoc analysis with Holm correction. **h**–**k** Differentially abundant ASVs (using Linear discriminant analysis effect size) from lungs of C57BL/6J mice (*n* = 6–10 per group, *N* = 1); **h** between NI and *Pb*K173 at day 5 p.i. **i** Between *Pb*ANKA and *Pb*K173 at day 5 p.i. **j** Between NI and *Pb*K173 at day 7 p.i. **k** Between *Pb*ANKA and *Pb*K173 at day 7 p.i. Source data are provided as a Source Data file.

We used two distinct rodent models of infection recapitulating MA-ARDS in humans^13^, either by infecting C57BL/6J mice with *P. berghei* K173 (*Pb* K173) (Supplementary Fig. 1a–d) or DBA/2 mice with *Pb* ANKA (Supplementary Fig. 1e–g)^24,25^, as well as a rodent model of cerebral malaria (eCM), by infecting C57BL/6J mice with *Pb* ANKA^26^ (Supplementary Fig. 1h–k). The two models of lung injury produce histopathological features very similar to those of human MA-ARDS. These include the expansion of the alveolar septae by parasitized RBCs and leukocytes, intra-alveolar haemorrhage and pulmonary oedema as measured by increased oedematous fluid content in the lungs^13^ (Supplementary Fig. 1c,d and g). In both rodent models of MA-ARDS, we see the onset of symptoms around days 6–7 post-infection with *Plasmodium* parasites with a significant decrease in oxygen saturation levels (SpO_2_, <90%) (Fig. 1a, b).

Our initial aim was to understand whether alterations in the microbiota composition in the lung are associated with the development of MA-ARDS in mice. We started by quantifying the number of colony-forming units (CFUs) of bacteria in the lungs of mice that die of MA-ARDS, when compared to those that die of eCM and non-infected (NI) mice. To do this, we plated lung extracts under sterile conditions on days 3 and 5 (prior to the onset of MA-ARDS and eCM) as well as 7 (onset of MA-ARDS and eCM) post infection (p.i.) of C57BL/6J mice infected with either *Pb* K173 or *Pb* ANKA parasites. We observed that on day 3 p.i. there is no difference in the number of CFUs of bacteria between any of the groups (Fig. 1c). However, there was a 2–3 fold increment in the number of CFUs of bacteria in the lungs of *Pb* K173-infected mice at day 5 p.i. (Fig. 1c, *P* = 0.004) that further increased by 9–10 fold at day 7 p.i. (Fig. 1c, *P* < 0.001), when compared to *Pb* ANKA-infected mice and non-infected (NI) mice.

We then investigated the microbial composition in the lungs of *Pb* K173-infected C57BL/6J mice compared to both *Pb* ANKA-infected mice and NI controls at days 5 and 7 p.i. by performing bacterial 16S rRNA gene sequencing. Relative abundance of bacterial families showed that C57BL/6J mice infected with *Pb* K173 exhibited altered lung microbiota composition compared to *Pb* ANKA-infected mice or NI controls both at day 5 (Fig. 1d) and day 7 p.i. (Supplementary Fig. 2a). Principal coordinate analysis (PCoA) showed that the lung microbiome of *Pb* K173-infected mice was significantly distinct from *Pb* ANKA-infected mice and NI controls both at days 5 (Fig. 1e, Supplementary Table 1) and 7 p.i. (Supplementary Fig. 2b, Supplementary Table 1). Reagent-driven bacterial DNA contamination is a common problem while doing 16S sequencing-based microbiome analysis from low bacterial biomass samples such as the lung. To mitigate this and to ensure the robustness of the conclusions, we sequenced blanks as a negative control, these were negative controls that were empty Eppendorf tubes processed identically for DNA purification, downstream processes and sequencing performed but without lung tissue added. We sequenced negative controls in parallel with our experimental samples to reduce any possibility of contamination. These are blank Eppendorf tubes processed with the exact same DNA extraction kits as the biological samples. Following the purification of DNA from the mock sample, sequencing was performed on the same sequencing run as the tissue samples. To demonstrate clearly that the blank-derived data (reagents only) are different from the data obtained from tissue samples, blank samples were compared to lung samples to the PCoA (Principal Coordinate Analysis) (Supplementary Fig. 8). The PCoA analysis here supports a limited effect of reagent contaminants. Indeed, the first component seems to be driven by K173 vs. NI/ANKA groups and no blank effect is visible. The blank samples segregate clearly away from the tissue samples in the second dimension. The *Pb* K173 lung microbiome is also associated with a decrease of microbial ASV (Amplicon Sequence Variant) diversity (Fig. 1 f–g, and Supplementary Fig. 2c). Notably, C57BL/6J mice infected with *Pb* ANKA, the experimental model of eCM (Supplementary Fig. 1h–k), had neither increase in CFUs nor changes in the lung microbiota throughout infection, when compared to NI mice (Fig. 1c–g). We then wanted to determine the differentially abundant bacterial ASVs (using Linear discriminant analysis effect size) from the lungs of C57BL/6J mice. There was an increase in the relative abundance of *Escherichia* and *Staphylococcaceae* compared to the controls both at day 5 and day 7 p.i. (Fig. 1h–k) and a relative decrease in the relative abundance of many other bacterial families (Fig. 1h–k). The relative increase of bacterial families like *Escherichia* and *Staphylococcaceae* in the lungs has also been recently reported for other pulmonary disorders^27,28^, thereby suggesting their potential involvement in the pathogenesis of MA-ARDS. Of additional interest, the gut microbiota is altered in both MA-ARDS and eCM infection models (Supplementary Fig. 2e, f), which is consistent with previous reports^18,19^, although such changes depend on the host’s genetic background^18^. Together, our data support the hypothesis that *Plasmodium* infection alters, both qualitatively and quantitatively, the microbial communities in the lung and that the observed alterations in the lung microbiota composition associates with the development of MA-ARDS.

### iRBCs sequestration in the lungs mediates microbiota dysbiosis and promotes MA-ARDS

**Fig. 2:**
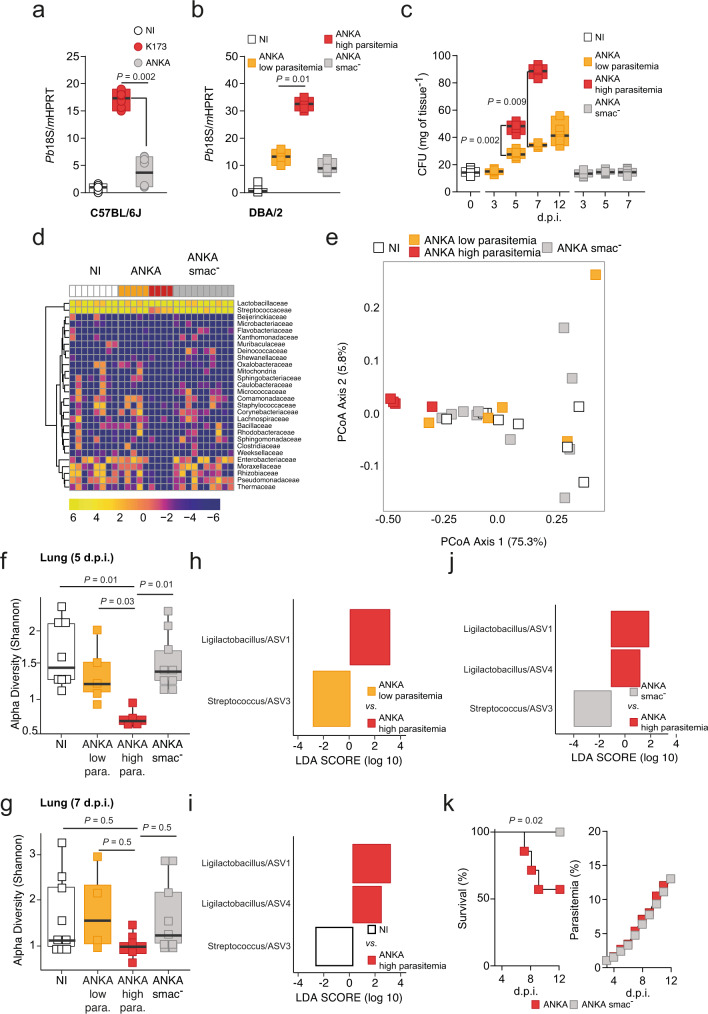
iRBCs sequestration in the lungs mediates microbiota dysbiosis and promotes MA-ARDS. **a** Parasite sequestration (Kruskal–Wallis) in the lungs of C57BL/6J mice on day 5 p.i. with *Pb* K173 or *Pb* ANKA and non-infected (NI) controls (*n* = 8 per group; *N* = 2). **b** Parasite sequestration (Kruskal–Wallis) in the lungs of DBA/2 mice on day 5 p.i. with Pb ANKA high parasitemia and *Pb* ANKA low parasitemia, *Pb* ANKA*smac-* and NI controls (*n* = 7–8 per group; *N* = 2). **c** Time course measurement of CFUs (Kruskal–Wallis) in the lungs of DBA/2 mice infected with *Pb* ANKA MA-ARDS, *Pb* ANKA no MA-ARDS, *Pb* ANKA*smac-* at days 3, 5, 7 and 12 p.i. and non-infected (NI) controls (*n* = 6–10 per group; *N* = 2). (**a**–**c**) Data are represented as floating bar plots (minimum to maximum) with line at the middle representing median. **d** Heatmap of relative abundance of major microbial families in the lungs of DBA/2 mice 5 days p.i. with *Pb* ANKA high parasitemia, *Pb* ANKA low parasitemia, *Pb* ANKA*smac-* and NI controls (*n* = 8–10 per group; *N* = 1). **e** Beta diversity analysis of lung microbial communities of DBA/2 mice 5 days p.i. with *Pb* ANKA high parasitemia, *Pb* ANKA low parasitemia, *Pb* ANKA*smac-* and NI controls (*n* = 8–10 per group; *N* = 1). **f**, **g** Alpha diversity (for group comparison, we used Kruskal–Wallis followed by two-sided Mann–Whitney post hoc analysis with Holm correction) of lung microbial composition of DBA/2 mice at **f** day 5 p.i. with *Pb* ANKA high parasitemia, *Pb* ANKA low parasitemia, *Pb* ANKA*smac-* and NI controls; or **g** day 7 p.i. with *Pb* ANKA high parasitemia, *Pb* ANKA low parasitemia, *Pb* ANKA*smac-* and NI controls (*n* = 8-10 per group; *N* = 1). Data are represented as boxplots with whiskers. The lower and upper hinges represent the 1st and third quartiles. The upper whisker extends to the largest value but no further than 1.5 * inter-quartile range (IQR). The lower whisker extends to the smallest value or most 1.5 * IQR. The Centre hinge is the second quartile. Differentially abundant ASVs (using Linear discriminant analysis Effect Size) from lungs of DBA/2 mice comparing (**h**) *Pb* ANKA high parasitemia and *Pb* ANKA low parasitemia at day 5 p.i.; **i** NI and *Pb* ANKA high parasitemia at day 5 p.i.; and **j** *Pb* ANKA*smac*- and *Pb* ANKA high parasitemia (*n* = 4–9 per group; *N* = 1) at day 5 p.i. **k** Survival (left panel, Log-rank Mantel-Cox) and parasitemia (right panel, mean ± s.e.m.; linear regression) following infection of DBA/2 mice with either *Pb* ANKA or *Pb* ANKA*smac-* (*n* = 8 per group; *N* = 2) parasites. Source data are provided as a Source Data file.

Adherence of *Plasmodium*-infected red blood cells (iRBCs) to the vascular endothelium, referred to as sequestration, is a crucial event for the onset of malaria pathology^29,30^. We observed that on day 5 p.i. there was increased parasite sequestration in the lungs of *Pb* K173-infected mice, when compared to *Pb* ANKA-infected mice and NI controls (Fig. 2a). Infection of DBA/2 mice with *Pb* ANKA is another model of MA-ARDS, leading to lung pathology in ~50% of the mice (Supplementary Fig. 1 e–g). We observed that there was a correlation between high levels of parasitemia at day 5 p.i. and mice that will succumb to MA-ARDS (Supplementary Fig. 1l). Accordingly, on day 5 p.i we observed an accumulation of iRBCs in the lungs of DBA/2 mice infected with *Pb* ANKA, which correlated with increased parasitemia and hence, the development of MA-ARDS (Fig. 2b). Next, we also observed an increase in CFUs of bacteria in the lung, seen on day 5 (prior to the onset of MA-ARDS) as well as day 7 (onset of MA-ARDS) following infection of DBA/2 mice with *Pb* ANKA parasites but not at day 3 p.i. (Fig. 2c). Importantly, there is a clear segregation in the lung bacterial burden at both days 5 and 7 p.i., with 50% and 60% of DBA/2 mice exhibiting increasing bacterial burden with the progression of the infection, respectively (Fig. 2c). In the remaining mice, the bacterial burden did not increase to the levels observed in those that will succumb to MA-ARDS (Fig. 2c). This is also the case of DBA/2 mice surviving infection with *Pb* ANKA analysed at day 12 p.i. (Fig. 2c), where bacterial burden in the lungs remains roughly at the same levels as those segregating at earlier time points.

Bacterial family abundance analysis showed that DBA/2 mice infected with *Pb* ANKA that will die of MA-ARDS (high parasitemia) have significant changes in the lung microbiome compared to mice that are protected, with the latter being similar to NI mice both at day 5 (Fig. 2d) and day 7 p.i. (Supplementary Fig. 3a). In addition, principal coordinate analysis (PCoA) showed that the lung microbiome of the mice infected with *Pb* ANKA and suffering MA-ARDS was significantly distinct from that of mice infected with *Pb* ANKA but not showing MA-ARDS, and NI controls both at day 5 (Fig. 2e, Supplementary Table 2) and day 7 p.i. (Supplementary Fig. 3b, Supplementary Table 2). ASV diversity was decreased in DBA/2 mice infected with *Pb* ANKA and suffering MA-ARDS although this decrease was less pronounced at day 7 p.i. (Fig. 2f, g and Supplementary Fig. 3c, d). There was an increase in the relative abundance of *Ligilactobacillus* compared to the controls on day 5 (Fig. 2h, i) and *Escherichia* on day 7 (Supplementary Fig. 3e–g). Importantly, infection of DBA/2 mice with *Pb* ANKA parasites lacking the schizont membrane-associated cytoadherence (*smac*) protein (*Pb* ANKA*smac*-), which do not sequester to the lung^31^ (Fig. 2b), failed to promote lung dysbiosis (Fig. 2c–g and j and Supplementary Fig. 3a–d and g) and MA-ARDS (Fig. 2k, and Supplementary Fig. 3h), but not gut microbiota dysbiosis (Supplementary Fig. 3i). These data show that iRBCs sequestration is the basis of alterations in the host lung microbiota and the development of MA-ARDS.

### Host microbiota is a contributing factor for MA-ARDS

Having established that *Plasmodium*-driven alterations in the lung microbiota composition associated with the development of ARDS, we assessed whether the clinical outcome of infection could be modulated by controlling bacterial expansion.

**Fig. 3:**
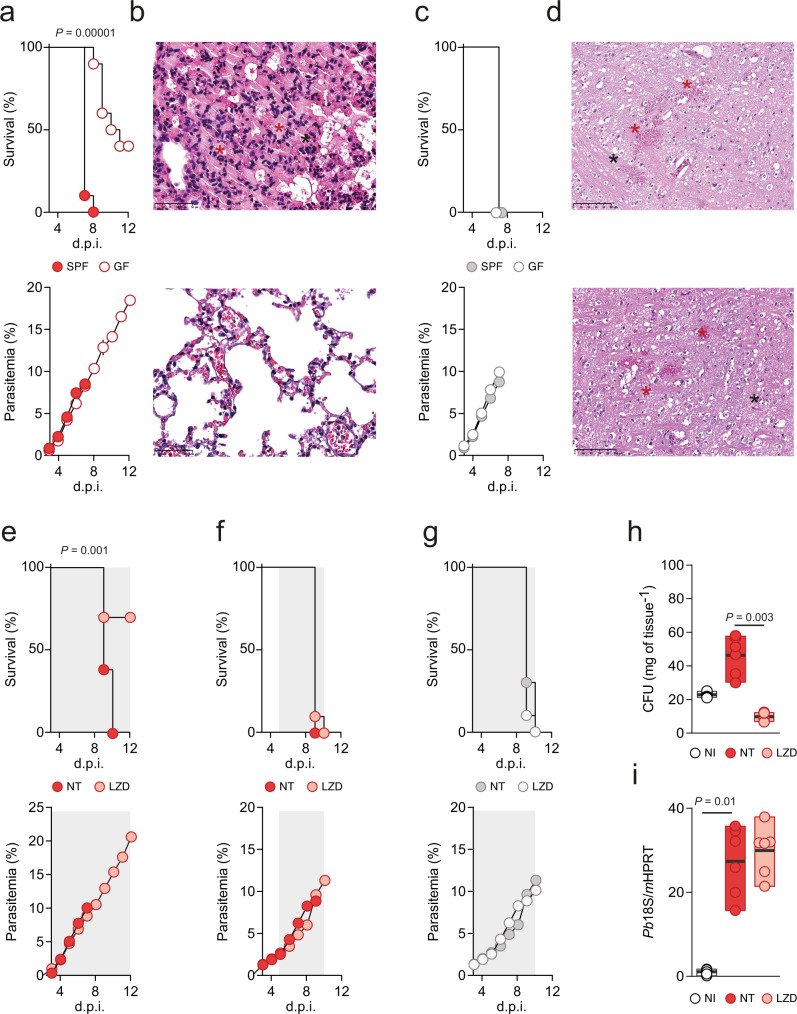
Host microbiota is a contributing factor for MA-ARDS. **a** Survival (upper panel, Log-rank Mantel-Cox) and parasitemia (lower panel, mean ± s.e.m.; linear regression) following *Pb* K173 infection of SPF or GF (*n* = 10 per group; *N* = 2) C57BL/6J mice. **b** Histological images of lungs of SPF (upper panel) and GF (lower panel) C57BL/6J mice following *Pb* K173 infection. Severe alveolar oedema (red asterisk) in the lung (collected at day 8 days p.i.) of SPF mice was observed. No changes were observed in the lung (collected at day 28 p.i.) of surviving GF mice (lower panel). Scale bar = 100 µm. **c** Survival (upper panel, Log-rank Mantel-Cox) and parasitemia (lower panel, mean ± s.e.m.; linear regression) following *Pb* ANKA infection of SPF or GF (*n* = 5 per group; *N* = 1) C57BL/6J mice. **d** Histological images of brains of SPF (upper panel) and GF (lower panel) C57BL/6J mice following *Pb* ANKA infection. In both groups in the mid-brain, marked haemorrhages (red asterisk) and grey matter vacuolization (black asterisk) were observed. Scale bar = 100 µm. **e** Survival (upper panel, Log-rank Mantel-Cox) and parasitemia (lower panel, mean ± s.e.m.; linear regression) following *Pb* K173 infection of linezolid treated C57BL/6J mice compared to non-treated, infected controls (*n* = 10 per group; *N* = 2). The shaded area represents the duration of antibiotic treatment starting at day 3 p.i. **f** Survival (upper panel, Log-rank Mantel-Cox) and parasitemia (lower panel, mean ± s.e.m.; linear regression) following *Pb* K173 infection of linezolid treated C57BL/6J mice compared to non-treated, infected controls (*n* = 10 per group; *N* = 2). The shaded area represents the duration of antibiotic treatment starting at 5 days p.i. **g** Survival (upper panel, Log-rank Mantel-Cox) and parasitemia (lower panel, mean ± s.e.m.; linear regression) following *Pb* ANKA infection of linezolid treated C57BL/6J mice compared to non-treated, infected controls (*n* = 10 per group; *N* = 2). **h** Total CFU’s (Kruskal–Wallis) in the lungs of C57BL/6 J mice 5 days p.i. with *Pb* K173 and treated with linezolid starting at 3 days p.i. compared to non-treated, *Pb* K173 infected and NI (*n* = 6 per group; *N* = 2) controls. **i** Parasite sequestration in the lungs of C57BL/6J mice (Kruskal–Wallis test followed by Wilcoxon post hoc analysis) 5 days p.i. with *Pb* K173 and treated with linezolid starting at 3 days p.i. compared to non-treated *Pb* K173 infected and NI (*n* = 6 per group; *N* = 2) controls. Data are represented as floating bar plots (minimum to maximum) with line at the middle representing median. Source data are provided as a Source Data file.

First, we compared the course of infection in germ-free (GF) versus specific pathogen-free (SPF) C57BL/6J mice infected with *Pb* K173. As before, all SPF mice infected with *Pb* K173 died on day 7 p.i. with MA-ARDS. Importantly, a significant proportion of *Pb* K173-infected GF mice did not develop MA-MARDS, and those that succumbed to MA-MARDS (60%) did so with a delay (9.8 vs 7.1 days, GF vs SPF mice, respectively, *P* = 0.002) (Fig. 3a, b and Supplementary Fig. 4a). All *Pb* ANKA-infected C57BL/6J mice died from eCM, irrespectively of being in SPF or GF conditions (Fig. 3c, d and Supplementary Fig. 4b).

In addition, we observed that administration of linezolid, an antibiotic used to treat lung-associated bacterial infections, prevented the development of MA-ARDS but not of eCM (Fig. 3e–g and Supplementary Fig. 4c–e). Even though linezolid does not confer protection once lung dysbiosis is established (such as on day 5 p.i., Fig. 3f and Supplementary Fig. 4d), initiation of treatment as late as day 3 after infection is sufficient to protect mice from MA-ARDS (Fig. 3e and Supplementary Fig. 4c). Importantly, linezolid-elicited protection is associated with lower bacterial load in the lung (Fig. 3h), without impacting systemic parasite burden (parasitemia) (Fig. 3e) or parasite sequestration. (Fig. 3i). Together, our data using GF and antibiotic-treated mice show that host microbiota is a contributing factor for MA-ARDS. However, the fact that 60% of GF mice still die with MA-ARDS, albeit with a delay, suggests that additional host factors contribute to the establishment of MA-ARDS and warrants further investigation.

### Increased IL-10 levels mediate bacterial expansion in the lung

**Fig. 4:**
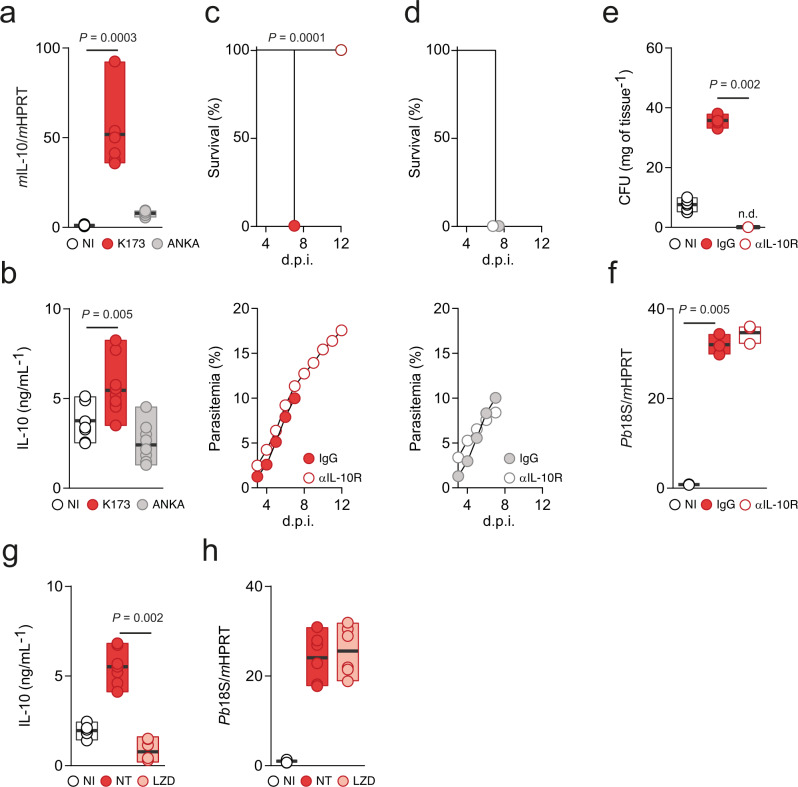
Increased IL-10 levels in the lungs mediate bacterial expansion in lung. **a** Total mRNA (Kruskal–Wallis) and **b** protein levels (Kruskal–Wallis test followed by Wilcoxon post hoc analysis) of IL-10 in the lungs of C57BL/6J mice 5 days p.i. with *Pb* K173 (*n* = 6; *N* = 2) or *Pb* ANKA (*n* = 6; *N* = 2) compared to NI controls (*n* = 6; *N* = 2)**. c** Survival (upper panel, Log-rank Mantel-Cox) and parasitemia (lower panel, mean ± s.e.m.; linear regression) following *Pb* K173 infection of IgG (*n* = 10; *N* = 2) or αIL-10R neutralizing antibody (*n* = 10; *N* = 2) treated C57BL/6J mice. **d** Survival (upper panel, Log-rank Mantel-Cox) and parasitemia (lower panel, mean ±  s.e.m.; linear regression) following *Pb* ANKA infection of IgG (*n* = 10; *N* = 2) or αIL-10R neutralizing antibody (*n* = 10; *N* = 2) treated C57BL/6J mice. **e** Total CFUs (Kruskal–Wallis test followed by Wilcoxon post hoc analysis) in the lungs of C57BL/6J mice 5 days p.i. with *Pb* K173 (*n* = 6; *N* = 2) treated with αIL-10R neutralizing antibody and compared to IgG treated (*n* = 6; *N* = 2) and NI (*n* = 6; *N* = 2) controls (n.d. = not detected). **f** Parasite sequestration (Kruskal–Wallis test followed by Wilcoxon post hoc analysis) in the lungs of C57BL/6J mice 5 days p.i. with *Pb* K173 (*n* = 6; *N* = 2) treated with αIL-10R neutralizing antibody and compared to IgG treated (*n* = 6; *N* = 2) and NI (*n* = 6; *N* = 2) controls. **g** IL-10 protein levels (Kruskal–Wallis test followed by Wilcoxon post hoc analysis) in the lungs of C57BL/6J mice 5 days p.i. with *Pb* K173 treated with linezolid starting 3 days p.i. (*n* = 4; *N* = 1) compared to NI (*n* = 4; *N* = 1) and non-treated, *Pb* K173 (*n* = 4; *N* = 1) infected controls. **h** Parasite sequestration (Kruskal–Wallis test followed by Wilcoxon post hoc analysis) in the lungs of C57BL/6J mice 5 days p.i. with *Pb* K173 treated with linezolid starting 3 days p.i. (*n* = 4; *N* = 1) compared to NI (*n* = 4; *N* = 1) and non-treated, *Pb* K173 (*n* = 4; *N* = 1) infected controls. **a**, **b**, **e**–**h** Data are represented as floating bar plots (minimum to maximum) with line at the middle representing median. Source data are provided as a Source Data file.

We next sought to elucidate the effector mechanisms by which iRBC sequestration would trigger altered microbial load and composition and the onset of MA-ARDS. Malaria is a multifactorial disease where both parasite and host factors contribute to the establishment of pathology. Sequestration of iRBCs is known to elicit a strong inflammatory response^32,33^. Thus, we assessed the levels of inflammatory mediators systemically and in the lungs. *Pb* K173-infected C57BL/6J mice (MA-ARDS) exhibited significantly altered levels of several circulating inflammatory modulators. In addition to VEGF, which is critical for the development of MA-ARDS^25^, the levels of IFN-γ and of the IFN-γ-induced molecules MCP-1 and IP-10, TNF, IL-6, and the anti-inflammatory cytokines LIF and IL-10 were increased when compared to NI mice, as well as to *Pb* ANKA-infected mice (eCM) (Supplementary Fig. 5a). One of the most differentially expressed circulating cytokines observed in this analysis was IL-10. We confirmed increased IL-10 levels in the lung of *Pb* K173-infected C57BL/6J mice, both at the mRNA and at the protein levels (Fig. 4a, b). IL-10 is a pleiotropic cytokine with broad, non-redundant immunosuppressive functions, particularly at mucosal sites, such as the intestine and lung, and is associated with tissue protection by controlling harmful immunopathology^34^. However, the local role of IL-10 during lung infections can be contextual, as IL-10 has also been implicated in increased susceptibility to some bacterial infections^35,36^. Notably, treatment of *Pb* K173-infected C57BL/6J mice with an IL-10R-neutralizing antibody (αIL-10R) conferred complete protection from MA-ARDS, as compared to isotype-treated mice (Fig. 4c and Supplementary Fig. 5b), while having no impact on eCM development (Fig. 4d and Supplementary Fig. 5c). Importantly, the protection conferred by the αIL-10R antibody is associated with decreased bacterial load (Fig. 4e) without affecting iRBC sequestration in the lungs (Fig. 4f). Conversely, antibiotic treatment resulted in a decrease in local IL-10 production (Fig. 4g), again without affecting iRBC sequestration in the lungs (Fig. 4h), implying a self-amplifying cycle between IL-10 and lung bacteria expansion leading to MA-ARDS.

### αβ T cells producing IL-10 promote altered bacterial colonization in the lung and MA-ARDS

**Fig. 5:**
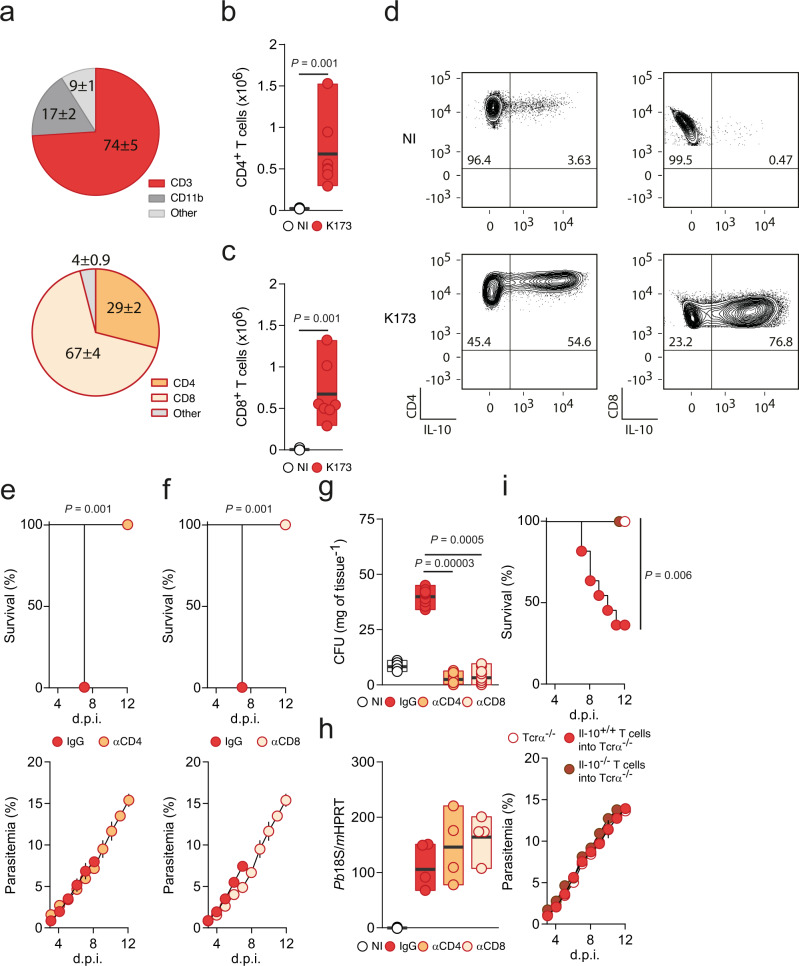
αβ T cells producing IL-10 promote altered bacterial colonization in the lung and MA-ARDS. **a** Average percentage of different immune cells (upper pie chart, the gating was done on live CD45^+^ cells) and T cells (lower pie chart, the gating was done on live CD45^+^CD3^+^ cells) producing IL-10 in the lungs of C57BL/6J Vert-X IL-10^GFP^ reporter mice 5 days p.i. with *Pb* K173 (*n* = 6; *N* = 2). **b** Total CD4^+^ (*n* = 6; *N* = 2) and **c** CD8^+^ T (*n* = 6; *N* = 2) cells producing IL-10 in the lungs of C57BL/6J Vert-X IL-10^GFP^ reporter mice 5 days p.i. with *Pb* K173 compared to NI controls (*n* = 6; *N* = 2) (Two-sided Mann–Whitney test). **d** Representative FACS plots of CD4^+^ (left panel) and CD8^+^ (right panel) T cells producing IL-10 in the lung of C57BL/6J Vert-X IL-10^GFP^ reporter mice 5 days p.i. with *Pb* K173 compared to NI controls. The gating was done on live CD45^+^CD3^+^ cells. **e** Survival (upper panel, Log-rank Mantel-Cox) and parasitemia (lower panel, mean ± s.e.m.; linear regression) following *Pb* K173 infection of IgG (*n* = 10; *N* = 2) or αCD4 depleting antibody (*n* = 10; *N* = 2) treated C57BL/6J mice, starting at day 3 p.i. **f** Survival (upper panel, Log-rank Mantel-Cox) and parasitemia (lower panel, mean ± s.e.m.; linear regression) following *Pb* K173 infection of IgG (*n* = 10; *N* = 2) or αCD8 depleting antibody (*n* = 10; *N* = 2) treated C57BL/6J mice, starting at day 3 p.i. **g** Total CFUs (Kruskal–Wallis test followed by Wilcoxon post hoc analysis) in the lungs of C57BL/6J mice 5 days p.i. with *Pb* K173 treated with either αCD4 (*n* = 8; *N* = 2) or αCD8 (*n* = 8; *N* = 2) depleting antibody compared to IgG treated (*n* = 8; *N* = 2) and NI (*n* = 8; *N* = 2) controls. **h** Parasite sequestration (Kruskal–Wallis test followed by Wilcoxon post hoc analysis) in the lungs of C57BL/6J mice 5 days p.i. with *Pb* K173 treated with either αCD4 (*n* = 4; *N* = 1) or αCD8 (*n* = 4; *N* = 1) depleting antibody compared to IgG treated (*n* = 4; *N* = 1) and NI (*n* = 4; *N* = 1) controls. **i** Survival (upper panel, Log-rank Mantel-Cox) and parasitemia (lower panel, mean ± s.e.m.; linear regression) of control TCRα^-/-^ mice, or upon adoptive transfer of wild-type or IL-10^-/-^ T cells, infected with *Pb* K173 (*n* = 12; *N* = 2). **b**, **c**, **g**, **h** Data are represented as floating bar plots (minimum to maximum) with line at the middle representing median. Source data are provided as a Source Data file.

Next, we sought to identify the source of IL-10. To this end, we used the C57BL/6J Vert-X IL-10^GFP^ reporter mice^37^. Infection with *Pb* K173 resulted in a build-up of IL-10-producing leucocytes, mostly CD4^+^ and CD8^+^ αβ T cells, when compared to NI mice (Fig. 5a–d, Supplementary Fig. 6a). IL-10 production was specific to the lung, as it was barely detectable in the spleen (Supplementary Fig. 6a). Accordingly, depletion of either CD4^+^ or CD8^+^ T cells resulted in a significant decrease in IL-10 levels in the lung (Supplementary Fig. 6b) and full protection from MA-ARDS, without affecting parasitemia (Fig. 5e and f, Supplementary Fig. 6c). CD4^+^ or CD8^+^ αβ T cell depletion also significantly decreased the lung bacterial load, with no significant changes in *Plasmodium* iRBC sequestration (Fig. 5g, h). Transfer of wild type, but not of IL-10-deficient αβ T cells, resulted in MA-ARDS development in otherwise protected TCRα-deficient mice, which are only deficient in αβ T cells (Fig. 5i, Supplementary Fig. 6e). These data demonstrate that a specific inflammatory signature, mostly supported by local production of the anti-inflammatory cytokine IL-10 by αβ T cells, promotes alterations in the lung microbiota composition that ultimately leads to MA-ARDS.

## Discussion

The international campaign against malaria was a public health success story at the beginning of this millennium. In the first 15 years of this period, the total number of malaria cases and deaths were reduced by half. However, since 2015 such progress has stalled^5^, raising awareness of the fact that our ultimate goal—the eradication of malaria—will entail a multifaceted approach resting on an increased understanding of the intricate interactions between *Plasmodium* and its hosts. We now add to such efforts by identifying the host microbiota as a contributing factor in shaping the outcome of infection. Although several correlations between gut microbiota and *Plasmodium* infections have been previously shown^38,39^, our results provide additional evidence of a three-way axis between *Plasmodium*, the host and its microbiota. A pathological cycle triggered by the infectious agent (sequestration of *Plasmodium* iRBCs) is self-amplified by the cross-talk between the host’s response (increased IL-10 levels) and its microbiota (reduced lung microbiota control). In fact, iRBCs sequestration in the lung and concomitant local inflammatory changes have been previously proposed as the main cause of MA-ARDS in humans^40^. We now provide data, using distinct rodent models of infection, showing that this is unequivocally the case in mice.

Bacteraemia and pneumonia may contribute to death in patients with MA-ARDS^40^. Due to the clinical overlap between bacteraemia, pneumonia and severe malaria, the World Health Organization recommends the use of broad-spectrum antibiotics to be included in the treatment regiments of such malaria cases^41^. Our findings provide a possible rational for this empirical clinical intervention; antibiotic treatment controlling alterations in the microbiota composition of the lung is sufficient to prevent MA-ARDS. We propose that controlling the lung-colonizing microbiota is critical in acute respiratory pathology conditions, such as those resulting from infection, and may provide a critical time window for the administration of an effective anti-malarial treatment that may save the lives of thousands of individuals. Most importantly, such interventions may potentially be extended to ARDS in general as it was recently shown that increased lung bacterial burden is predictive of worse outcomes in ARDS-admitted patients^42^.

The study of the microbiome has yielded fascinating insights into the pathogenesis of several diseases. Bacterial density in the airways is low, when compared to that of the gut. Still, in the last decade, we have observed a number of studies reporting significantly alterations of the lung microbiota in several disease conditions, such as COPD, cystic fibrosis, asthma and lung cancer^21,22,43^. While such observations suggest that the lung microbiota influences both respiratory health and disease, the gut microbiota can also influence pulmonary immunity through what is commonly referred to as the gut–lung axis^28^. Indeed, gut-associated bacterial families have recently been reported to be over-represented in the lungs of ARDS patients with poorer outcomes^42^. Thus, either remote (gut) or local (lung) microbiota may contribute to the increase in bacterial burden, which we now report to be present in the lungs of mice before the onset of MA-ARDS. A question that need’s to be addressed in the future is the relative contribution of local (lung) and remote (lower gut) bacterial communities to lung inflammation and injury.

The seemingly surprising disease-promoting role of IL-10 in MA-ARDS may represent an imbalance between its cardinal role in preventing immunopathology during infection, and its concomitant inhibition of microbial control resulting in bacterial outgrowth and dissemination^34,44^. IL-10 is a cytokine with potent anti-inflammatory properties that plays a central and non-redundant role in limiting host immune responses to distinct danger signals, thereby preventing damage to the host and restoring tissue homeostasis. Indeed, dysregulation of IL-10 is frequently associated with enhanced immunopathology, as many of the severe complications observed in a wide range of infections (including malaria) result from excessive immune activation. However, our data show that blockage of IL-10 signalling fully protects mice from the onset of MA-ARDS, which is characterized by inflammatory injury to the alveolar capillary barrier both in mice and humans. We propose that this unexpected result is due to the fact that IL-10 may prevent appropriate bacterial control^44^, which ultimately causes an increase in the relative abundance of certain bacterial species. Indeed, we now show that MA-ARDS prevention by IL-10 signalling blockage is accompanied by a strong and significant decrease in bacterial load. A previous study highlighted the critical role of CD8^+^ T cells in mediating MA-ARDS^45^. Indeed, we see that CD8^+^ T cell depletion protects mice from MA-ARDS. However, here we provide novel evidence that IL-10 production by either CD4^+^ or CD8^+^ T cells is responsible for alterations in the microbiota composition of the lungs and the development of MA-ARDS. Most importantly, and in line with our data, elevated IL-10 levels in adults with *P. falciparum* malaria has prognostic significance for death in severe malaria cases without cerebral involvement^40^ and early measurement of IL-10 predicts a worse outcome of patients with non-malaria ARDS receiving extracorporeal membrane oxygenation^46^. The latter implies that our observations may extend to ARDS originating from various etiologies.

Overall, our mechanistic data using rodent models of MA-ARDS, together with the notion that lung bacterial burden predicted the clinical outcome of ARDS^42^, may prove useful in the clinical management of patients with respiratory life-threatening complications, such as those caused by *Plasmodium* but also influenza or corona viruses, and highlights that tailored, lung-microbiota targeting antibiotic treatment may constitute a critical tool in the clinical weaponry against ARDS.

## Methods

### Mice

Male C57BL/6J and DBA/2 mice were purchased from Charles River breeding laboratories and housed in the animal facilities at Instituto de Medicina Molecular João Lobo Antunes (iMM-JLA) and Instituto Gulbenkian de Ciência (IGC) in specific pathogen-free (SPF) conditions. Germ-free mice were generated at the germ-free breeding facility at IGC and housed in gnotobiotic conditions. C57BL/6J Vert-X IL-10^GFP^ reporter mice were obtained from the animal house at the Francis Crick Institute in London, United Kingdom and, like the TCRα-deficient mice (Jackson laboratories Stock No:004364), housed at the SPF facility of iMM-JLA. Animals were housed under the following conditions with 14 h light:10 h dark cycle, temperature 20–24 °C and relative humidity of 55 ± 10%.

Animal experiments were performed according to EU regulations and approved by the Órgão Responsável pelo Bem-estar Animal (ORBEA) of Instituto de Medicina Molecular and by the Direcção-Geral de Alimentação e Veterinária (Portugal),

For gut and lung microbiome analysis, unless otherwise stated, C57BL/6J mice were co-housed under specific pathogen-free conditions with 3 mice per cage in 10 different cages. After 2 weeks of co-housing mice were infected with different *Plasmodium* strains together with non-infected mice in each cage to account for any cage variability. For DBA/2 mice, 4 mice were co-housed together for 2 weeks, with 1 mice kept as sentinel to monitor microbiome variations due to bedding or cage effect.

### Parasites and infections

Male C57BL/6J and DBA/2 mice were infected, by intraperitoneal route, with 1 × 10^6^ luciferase-expressing *Plasmodium berghei* ANKA (GFP-Luc_schiz_, 1037cl1), *Plasmodium berghei* K173 (GFP-Luc_schiz_, 1006cl1) and *Plasmodium berghei* ANKA *smac-* (1242cl5m1cl1cl2) infected red blood cells (iRBC). Survival and parasitemia, assessed by Giemsa stained blood smears and expressed as percentage of iRBC, were monitored daily.

### Oxygen saturation measurements

Pulse oximeter (StarLife Sciences) was used to measure blood SpO2 on mice three, five and seven days post-infection. Mice were held by hand and the pulse oximeter clip was placed on the hind leg. Mice were held and covered by the light blocker fabric supplied by the manufacturer and held until calm (several seconds), before placing the probe on the hind leg over the skin near the femur bone. Three minute readings were taken from each mouse the average of 5–10 individual readings were calculated.

### Bacterial DNA isolation

Lungs and faecal pellets were harvested under sterile conditions and immediately snap frozen in liquid nitrogen. Samples were stored at −80 °C until processing for DNA isolation. Genomic DNA was extracted from lung using a commercial kit (DNeasy tissue kit; Qiagen, Germantown, MD) and from faecal pellets using QIAamp Fast DNA Stool Mini Kit (Qiagen). Lung samples were processed based on a modified protocol previously demonstrated to isolate bacterial DNA from lungs^27,47^. Briefly, lungs were subjected to bead beating for 1 min using 1 mm diameter silica beads (BioSpec Products, United States, catalogue number: 11079110z) in a MiniBeadBeater homogenizer (BioSpec Products) for 2 min. Lung samples were incubated overnight at 60 °C with 400 µl of Tissue lysis buffer and 50 µl of lysozyme included in the kit. Following this, DNA was extracted following instructions from the DNeasy tissue kit. DNA was used directly for 16S rRNA analysis.

### 16S rRNA analysis

16S rRNA gene amplification and sequencing were carried out at the Genomics Unit at IGC. The V4 region of the 16S rRNA gene was amplified in triplicate for each sample, using the primer pair F515/R806. PCRs were done under the following conditions: 3 min at 94 °C, 35 cycles of 60 s at 94 °C, 60 s at 50 °C, and 105 s at 72 °C, with an extension step of 10 min at 72 °C. Samples were then pair-end sequenced on an Illumina MiSeq Benchtop Sequencer (Illumina), following Illumina recommendations^48,49^.

Each lung sample was cleaned of *Plasmodium* parasite and host sequences using Kraken2. To do this, we first created a Kraken2 index consisting of the Bacteria and Archaea libraries plus the mouse genome (GRCm39) and the genomes of *Plasmodium berghei* ANKA (GCA_900002375.2) and K173 (GCA_900044334.1), all of which were downloaded from NCBI. The reads of each sample were then mapped against this database with Kraken2 and the script extract_kraken_reads.py (from KrakenTools; https://github.com/jenniferlu717/KrakenTools) was used to filter reads in order to keep only those mapping to Bacteria or Archaea for further analysis (Supplementary Table 3).

Illumina-sequenced paired-end fastq files were analysed using the DADA2 pipeline^50^. Fastq files were filtered and trimmed using the following standard filtering parameters maxN=0, maxEE=c(2,2), truncQ=2. We applied truncLen=c(240,150) for C57BL/6 J lung, truncLen=c(230,200) for DBA/2 Lung, truncLen=c(210,160) for C57BL/6J gut, truncLen=c(240,210) for DBA/2 gut datasets.

The data is merged into paired end reads, and used to infer sample composition and construct an amplicon sequence variants (ASV) table. Then, we removed the chimeras, and assigned to taxonomy using Silva version 138.1 training set^51,52^ (released March 7, 2021). We then remove ASVs corresponding to Archea, Chloroplast and Mitochondria. At this point, we were able to identify 2259 ASVs for C57BL/6J Lung, 2109 ASVs for DBA/2 Lung.

Contaminating sequences were identified and removed using decontam v1.12.0^53^, based on identities of ASVs from blanks. We used the prevalence test using the parameter threshold = 0.5, which identify as contaminants all sequences more prevalent in negative controls than in real samples. We identified and removed 101 sequence features as contaminants in C57BL/6J lung and 81 sequence features in DBA/2 lung.

All downstream analysis were performed on R (v4.0.2) (R CoreTeam, 2020). For data manipulation, we used the packages dplyr (v 1.0.5)^54^ and tidyverse (v1.3.0)^55^.

All reads identified as bacteria were kept. Then, we remove C57BL/6J lung samples under 6000 reads and DBA/2 Lung samples under 3000 reads. Consequently, our library size varies between 6097 and 114,667 for C57BL/6J Lung, from 3317 to 155,163 for DBA/2 Lung.

Then we rarefied the data using rrarefy (vegan v2.5.7)^56^ (Supplementary Fig. 7c and d). We compared the microbiome between experimental groups with Permutational Multivariate ANalysis Of Variance (PERMANOVA) using the function adonis (vegan v2.5.7) with 999 permutations. Dissimilarity distances (Bray-Curtis) were calculated using vegdist (vegan v2.5.7) then reduced to principal coordinates using betadisper (vegan v2.5.7) and finally plotted using ggplot2 (v3.3.3)^57^.

To find the differentially abundant ASVs, we transformed our data into a SummarizedExperiment object using SummarizedExperiment (v1.20.0)^58^, then we ran linear discriminant analysis effect size (LEfSe) algorithm (Kruskal–Wallis test, *p* < 0.05, LDA score > 2.0) using lefser (v1.0.0)^59^.

For visualization of the C57BL/6J heatmap we choose to display the bacterial families that have at least 10 reads per mouse in each experimental group (e.g., for a group of 9 mice, the number of reads needed to plot one family is 90). For visualization of the DBA/2 heatmap dataset, we removed bacterial families that showed no count (zero) across 22 of the mice. The heatmaps were generated using pheatmap (pheatmap v1.0.12)^60^. Diversity calculations were done at ASV levels using diversity function (vegan v2.5.7) for Shannon index and Chao1 (fossil v 0.4.0)^61^.

### Determination of bacterial colony-forming-units (CFUs)

Whole lung homogenates were prepared as described above, dissolved in 3 ml of sterile saline and filtered through a 100-micron filter (Corning, catalogue number: 352360). 100 µl from each of the lung homogenates was then plated on blood agar plates (using Columbia blood agar base, Oxoid, catalogue number: CM0331 with 5% sterile defibrinated blood) and incubated at 5% CO_2_ at 37 °C. CFUs were counted after 24–48 h.

### RNA isolation, cDNA and quantification of gene expression

Lungs were harvested under sterile conditions, placed in 1 ml PureZOL (Bio-Rad, catalogue number: 732-6890) and mechanically homogenized using 1 mm diameter silica beads (BioSpec Products, United States, catalogue number: 11079110z) in a MiniBeadBeater homogenizer (BioSpec Products) for 2 min. Two hundred (200) μl of the lysate was used to isolate total RNA using the NZY total RNA Isolation kit (NZYTech, catalogue number: MB13402) according to the manufacturer’s instructions. DNAse-treated RNA was reverse transcribed with random hexamers using NZY First-Strand cDNA Synthesis Kit (NZYTech, catalogue number: MB12501). Real-time PCR was performed on ABI 7500 or 7900HT systems (Applied Biosystems), using Universal SYBR Green Supermix (Bio-Rad, catalogue number: 172-5121) and the primers listed below. Relative gene expression was normalized to the geometric mean of the housekeeping gene hypoxanthine guanine phosphoribosyltransferase (Hprt) (ΔCt). Gene expression values were then calculated based on the ΔΔCt method, using the mean of the control group as the calibrator to which all other samples were compared.

Primers used were:

mHprt:

CATTATGCCGAGGATTTGGA; AATCCAGCAGGTCAGCAAAG

Pb18S rRNA:

AAGCATTAAATAAAGCGAATACATCCTTAC; GGAGATTGGT TTTGACGTTTATGTG

mIL-10:

CAGCCGGGAAGACAATAACT; GTTGTCCAGCTGGTCCTTTG

### Broncho alveolar lavage (BAL) collection

Mice were euthanized by administering ketamine (100 mg/mL solution of ketamine in sterile saline) intraperitoneally (IP) at a dose of 120 mg/kg. The trachea was then exposed through a midline incision and cannulated with a sterile 21-gauge T catheter (Venofix, catalogue number: 4056337). BAL was performed by instillation of two 0.5 ml aliquots of sterile saline into the right lung. The retrieved BAL fluid (∼0.8 ml) was used for further experiments.

### Measurement of cytokines and chemokines

BAL fluid harvested on day 5 p.i. from different rodent-parasite combinations was assayed for IL-10 levels by sandwich enzyme-linked immunosorbent assay (ELISA) method using a commercially available murine IL-10 capture kit (PeproTech, catalogue number: 900-K53) according to the manufacturer’s instructions. Circulating cytokine and chemokine levels were quantified in serum using Mouse cytokine 32-plex discovery assay by Eve Technologies (Canada).

### Mononuclear cell isolation from lungs

Lungs were harvested from C57BL/6J Vert-X IL-10^GFP^ reporter mice and washed with sterile 1× PBS and placed in Petri dishes with RPMI 1640 medium (3 ml) (Sigma-Aldrich, catalogue number: C5138). The whole lung was minced with forceps and scissors to 1 mm sized pieces and incubated with 3 ml of digestion medium (2 mg of Collagenase type IV, 3 ml of RPMI 1640 medium with Type IV DNase I (final concentration: 25units/ml, Sigma-Aldrich, catalogue number: C5138) at 37 °C under agitation (200 rpm) conditions for 45 min. Following that, tissue digestion was stopped by adding 1 ml of heat-inactivated FBS (Life Technologies, catalogue number: 10437-028). The cells were then dispersed with a 10 ml syringe (BD Biosciences, catalogue number: 301604) fitted with an 18gauge needle (10 times) (BD Biosciences, catalogue number: 305180) and filtered using a cell strainer (100μm, Corning, catalogue number: 352360). The cells were then centrifuged (Centrifuge 5810 Eppendorf) at 10 °C and 300 × *g* for 5 min. The supernatant was discarded and 1 ml of red blood cell lysis buffer (0.144 M NH_4_Cl, 0.0169 M TRIS base, pH 7.4) was added and incubated at room temperature for 1 min. The reaction was stopped by adding 10 ml of 1× PBS with 10% heat-inactivated FBS. Finally, the cells were centrifuged at 10 °C and 300 × *g* for 5 min and the pellet was resuspended in FACS buffer (PBS + 2%FCS) for further staining.

### Flow cytometry staining and antibodies

For direct multi-colour flow cytometry (LRS Fortessa X-20; BD Bioscience), cells were incubated for 30 min at 4 °C with the antibodies mentioned below. Live/dead cell discrimination was performed by staining with the Zombie Aqua Fixable Viability Kit (BioLegend, catalogue number: 423101). To block Fc receptors, a purified anti-mouse CD16/CD32 (eBioscience, catalogue number: 14-0161-86) was used.

CD19 APCCy7 (clone 6D5, Biolegend, catalogue number: 115530, 1:300), Ly6G PerCPCy5.5 (clone 1A8, Biolegend, catalogue number: 127616, 1:300), CD45 PEDazzle (clone 30-F11, Biolegend, catalogue number: 103146, 1:400), F4/80PECy7 (clone BM8, eBioscience, catalogue number:254801-82, 1:200), CD3 APC (clone 145-2c11, Biolegend, catalogue number: 100312, 1:100), CD11b Alexa700 (clone M170, Biolegend, catalogue number: 101222, 1:400), TCγδV421 (clone GL3, Biolegend, catalogue number: 118120, 1:100), CD4 BV605 (clone RM4-5, Biolegend, catalogue number: 100548, 1:300), CD8 BV711 (clone 53-6.7, Biolegend, catalogue number: 100748, 1:300) and NK1.1 PE (clone PK136, Biolegend, catalogue number: 557391, 1:300). IL-10^GFP^ production was measured in the FITC channel.

### Antibody treatments

α-IL-10R rat monoclonal antibody (clone 1B1.2), anti-CD4 (clone YTA 3.1) and anti-CD8 (clone YTS 156/169) were kindly provided by Dr. Luis Graça^62^. IL-10 signalling was blocked by injecting, by intraperitoneal route, 100 µg of α-IL-10R monoclonal antibody at days 0, 2 and 4 post infection. Control groups were injected with rat immunoglobulin in parallel (Rat IgG2b Biolegend, catalogue number: 400602). For in vivo CD4+ and CD8+ T cell depletion experiments, mice were injected intraperitoneally with 50 µg of each antibody individually or the corresponding isotype control (rat IgG2b Biocell, catalogue number: BE0117) at days 0 and 3 post infection.

### T cell transfer

Lymphocytes were isolated from spleens and lymph nodes of C57BL/6 J WT and IL10^−/−^ mice. T cells were separated using magnetic-activated cell sorting (MACS). Briefly, lymphocytes were incubated in MACS buffer (1xPBS (Sigma-Aldrich, catalogue number: F9665) with 0.5% FBS (Sigma-Aldrich, catalogue number: F9665) and 2 mM EDTA (Sigma-Aldrich, catalogue number: 03690) with anti-Thy1.2-APC antibody (CD90.2) (clone 53-2.1, Biolegend, catalogue number: 140312) for 10 min, washed and subsequently incubated with anti-APC microbeads (Milteny Biotec, catalogue number: 130-090-855) for 20 min at 4 °C. The cell suspension was filtered and APC/Thy1.2 positive cells (CD4^+^ and CD8^+^) were collected using an autoMACS separator (Milteny Biotec). Live cells obtained from the positive fraction were counted in trypan blue solution (Sigma-Aldrich, catalogue number: T8154) using a hemocytometer (Marienfeld, catalogue number: 0640010). An aliquot of the cells was analysed by flow cytometry in a Fortessa X20 to determine the distribution of CD4^+^ and CD8^+^ cells, using anti-CD4 PeCy7 (clone RM4-5, BioLegend, catalogue number: 100528) and anti-CD8 FITC (clone 53-6.7, catalogue number: 100706) antibodies. 50–60% and 40–50% of the cells were CD4^+^ and CD8^+^, respectively. 2.5 × 10^7^ live cells in 200 µL PBS were injected intravenous in TCRα^−/−^ recipient mice.

### Antibiotic administration

Mice received 25 mg/kg of the antibiotic linezolid (Sigma-Aldrich PZ-0014) twice daily by oral gavage starting at either day 3 or day 5 p.i.

### Histopathology

Mice were euthanized with CO_2_ narcosis, necropsy was performed and brains and lungs were collected and fixed in formalin. Heads were decalcified for 3 h using RDO (Rapid Decalcifying) solution, processed for paraffin embedding, sectioned at 3 μm and stained with hematoxylin and eosin (H&E) for routine histopathological analysis. Evidence for experimental cerebral malaria, including cerebral oedema and multifocal haemorrhage, were screened (0—absent, 1—minimal, 2—mild, 3— moderate, 4—severe) in all animals. Pulmonary oedema was scored as a measure of increased oedematous fluid content in the lungs of mice that succumb to MA-ARDS.

### Statistics

Significance was calculated using different tests on the GraphPad (Prism) 5.0 software. Statistical differences between the 2 groups were analysed using the non-parametric two-tailed Mann–Whitney test and in case of groups involving more than 2 datasets the data was analysed by Kruskal–Wallis. Survival and parasitaemia curves were analysed using the Log-rank Mantel-Cox Chi-squared test and linear regression curve. Time course measurement of oxygen saturation levels (SpO2) as determined by pulse-oximetry was analysed using Kruskal–Wallis test. Significance was considered for *P* values below 0.05. Biological replicates (*n*) indicated in figure legends refer to the number of mice. Sample sizes were chosen on the basis of historical data; no statistical methods were used to predetermine sample size. For diversity data, group comparisons were performed using Kruskal–Wallis test with kruskal.test function (stats v4.1.0) followed by two-sided Mann–Whitney post hoc analysis using wilcox.test function (stats v4.1.0). We used Holm correction for multiple comparisons.

### Reporting summary

Further information on research design is available in the Nature Research Reporting Summary linked to this article.

## Supplementary information

Supplementary Information

Reporting Summary

## Data Availability

Data generated for this manuscript is available. 16S rRNA amplicon sequencing data are deposited in the SRA database with the BioProject accession number PRJNA746689. The link for the sequencing data is https://www.ncbi.nlm.nih.gov/sra/PRJNA746689. Source data are provided with this paper.

## Source data

Source Data

## Code availability

All R code is available at https://github.com/lonej/ARS_Lung_Microbiome.

## References

[1] Matthay MA (2018). Acute respiratory distress syndrome. Nat. Rev. Dis. Prim..

[2] Short KR, Kroeze EJBV, Fouchier RAM, Kuiken T (2014). Pathogenesis of influenza-induced acute respiratory distress syndrome. Lancet Infect. Dis..

[3] Rubenfeld GD (2005). Incidence and outcomes of acute lung injury. N. Engl. J. Med..

[4] Villar J (2011). The ALIEN study: incidence and outcome of acute respiratory distress syndrome in the era of lung protective ventilation. Intensive Care Med..

[5] World Health Organization (WHO), *World Malaria Report* (2021).

[6] Hill AVS (1991). Common West African HLA antigens are associated with protection from severe malaria. Nature.

[7] Driss A (2011). Genetic polymorphisms linked to susceptibility to malaria. Malar. J..

[8] Kyriacou HM (2006). Differential var gene transcription in Plasmodium falciparum isolates from patients with cerebral malaria compared to hyperparasitaemia. Mol. Biochem. Parasitol..

[9] Kalmbach Y (2010). Differential *var* gene expression in children with malaria and antidromic effects on host gene expression. J. Infect. Dis..

[10] Dondorp AM (2008). The relationship between age and the manifestations of and mortality associated with severe malaria. Clin. Infect. Dis..

[11] Laishram DD (2012). The complexities of malaria disease manifestations with a focus on asymptomatic malaria. Malar. J..

[12] Mazhar F, Haider N (2016). Respiratory manifestation of malaria: an update. Int. J. Med. Res. Heal. Sci..

[13] Van den Steen PE (2013). Pathogenesis of malaria-associated acute respiratory distress syndrome. Trends Parasitol..

[14] Mohan A, Sharma SK, Bollineni S (2008). Acute lung injury and acute respiratory distress syndrome in malaria. J. Vector Borne Dis..

[15] Ho, J. T. K., Chan, G. C. F. & Li, J. C. B. Systemic effects of gut microbiota and its relationship with disease and modulation. *BMC Immunol.* **16**, 21 (2015). 10.1186/s12865-015-0083-2 PMC4404277 25896342

[16] Wang B, Yao M, Lv L, Ling Z, Li L (2017). The human microbiota in health and disease. Engineering.

[17] Carding S, Verbeke K, Vipond DT, Corfe BM, Owen LJ (2015). Dysbiosis of the gut microbiota in disease. Microb. Ecol. Health Dis..

[18] Taniguchi, T. et al. *Plasmodium berghei* ANKA causes intestinal malaria associated with dysbiosis. *Sci. Rep*. **5**, 15699 (2015). 10.1038/srep15699 PMC4621605 26503461

[19] Mooney JP (2015). Inflammation-associated alterations to the intestinal microbiota reduce colonization resistance against non-typhoidal Salmonella during concurrent malaria parasite infection. Sci. Rep..

[20] Lozupone CA, Stombaugh JI, Gordon JI, Jansson JK, Knight R (2012). Diversity, stability and resilience of the human gut microbiota. Nature.

[21] O’Dwyer DN, Dickson RP, Moore BB (2016). The lung microbiome, immunity, and the pathogenesis of chronic lung disease. J. Immunol..

[22] Salami O, Marsland BJ (2015). Has the airway microbiome been overlooked in respiratory disease?. Genome Med..

[23] Jin C (2019). Commensal microbiota promote lung cancer development via γδ T cells. Cell.

[24] Hee L (2011). Reduced activity of the epithelial sodium channel in malaria-induced pulmonary oedema in mice. Int. J. Parasitol..

[25] Epiphanio S (2010). VEGF promotes malaria-associated acute lung injury in mice. PLoS Pathog..

[26] de Souza JB, Hafalla JCR, Riley EM, Couper KN (2010). Cerebral malaria: why experimental murine models are required to understand the pathogenesis of disease. Parasitology.

[27] Ashley, S. L. et al. Lung and gut microbiota are altered by hyperoxia and contribute to oxygen-induced lung injury in mice. *Sci. Transl. Med*. **12**, eaau9959 (2020). 10.1126/scitranslmed.aau9959 PMC7732030 32801143

[28] Ubags NDJ, Marsland BJ (2017). Mechanistic insight into the function of the microbiome in lung diseases. Eur. Respir. J..

[29] Miller LH, Ackerman HC, Su X, Wellems TE (2013). Malaria biology and disease pathogenesis: insights for new treatments. Nat. Med..

[30] Hanson J (2012). Relative contributions of macrovascular and microvascular dysfunction to disease Severity in *Falciparum Malaria*. J. Infect. Dis..

[31] Fonager J (2012). Reduced CD36-dependent tissue sequestration of Plasmodium-infected erythrocytes is detrimental to malaria parasite growth in vivo. J. Exp. Med..

[32] Anstey NM (2007). Lung injury in *Vivax Malaria*: pathophysiological evidence for pulmonary vascular sequestration and posttreatment alveolar-capillary inflammation. J. Infect. Dis..

[33] Amante FH (2010). Immune-mediated mechanisms of parasite tissue sequestration during experimental cerebral malaria. J. Immunol..

[34] Ouyang W, O’Garra A (2019). IL-10 family cytokines IL-10 and IL-22: from basic science to clinical translation. Immunity.

[35] Sun K, Torres L, Metzger DW (2010). A detrimental effect of interleukin-10 on protective pulmonary humoral immunity during primary influenza A virus infection. J. Virol..

[36] van der Sluijs KF (2004). IL-10 is an important mediator of the enhanced susceptibility to *Pneumococcal Pneumonia* after influenza infection. J. Immunol..

[37] Madan R (2009). Nonredundant roles for B cell-derived IL-10 in immune counter-regulation. J. Immunol..

[38] Villarino NF (2016). Composition of the gut microbiota modulates the severity of malaria. Proc. Natl Acad. Sci. USA.

[39] Yooseph S (2015). Stool microbiota composition is associated with the prospective risk of *Plasmodium falciparum* infection. BMC Genomics.

[40] Taylor WRJ, Hanson J, Turner GDH, White NJ, Dondorp AM (2012). Respiratory manifestations of malaria. Chest.

[41] World Health Organization. Guidelines for the treatment of Malaria. *World Malar. Rep*. (2015).

[42] Dickson RP (2020). Lung microbiota predict clinical outcomes in critically Ill patients. Am. J. Respir. Crit. Care Med..

[43] Jin C (2019). Commensal microbiota promote lung cancer development via γδ T cells. Cell.

[44] Peñaloza HF (2016). Opposing roles of IL-10 in acute bacterial infection. Cytokine Growth Factor Rev..

[45] Claser C (2019). Lung endothelial cell antigen cross-presentation to CD8+T cells drives malaria-associated lung injury. Nat. Commun..

[46] Liu C-H (2017). Early measurement of IL-10 predicts the outcomes of patients with acute respiratory distress syndrome receiving extracorporeal membrane oxygenation. Sci. Rep..

[47] Mason KL (2012). Candida albicans and bacterial microbiota interactions in the cecum during recolonization following broad-spectrum antibiotic therapy. Infect. Immun..

[48] Caporaso JG (2011). Global patterns of 16S rRNA diversity at a depth of millions of sequences per sample. Proc. Natl Acad. Sci. USA.

[49] Caporaso JG (2012). Ultra-high-throughput microbial community analysis on the Illumina HiSeq and MiSeq platforms. ISME J..

[50] Callahan B (2016). DADA2: High resolution sample inference from amplicondata. Nat Methods.

[51] Yilmaz P (2014). The SILVA and “All-species Living Tree Project (LTP)” taxonomic frameworks. Nucleic Acids Res.

[52] Quast C (2013). The SILVA ribosomal RNA gene database project: improved data processing and web-based tools. Nucleic Acids Res,.

[53] Davis, N. M., Proctor, D. M., Holmes, S. P., Relman, D. A. & Callahan, B. J. Simple statistical identification and removal of contaminant sequences in marker-gene and metagenomics data. *Microbiome* **6**, 226 (2018). 10.1186/s40168-018-0605-2 PMC6298009 30558668

[54] Wickham H (2011). The split-apply-combine strategy for data analysis. J. Stat. Softw..

[55] Wickham H (2019). Welcome to the Tidyverse. J. Open Source Softw..

[56] Oksanen, J. et al. *Package ‘vegan’ Title Community Ecology Package Version 2.5-7* (2020).

[57] Wickham, H. & Wickham, H. in *ggplot2* 9–26 (Springer New York, 2009).

[58] Morgan, M., Obenchain, V., Hester, J., Pagès, H. & Bioconductor, M. *Encoding UTF-8*. (2020).

[59] Khleborodova, A. lefser: R implementation of the LEfSE method for microbiome biomarker discovery. R package version 1.0.0. (2020).

[60] Kolde, R. pheatmap: Pretty Heatmaps. R package version 1.0.12 (2019).

[61] Vavrek, M. J. Fossil: Palaeoecological and palaeogeographical analysis tools. *Palaeontologia Electronica* **14**, 16 (2011).

[62] Agua-Doce A (2018). Route of antigen presentation can determine the selection of Foxp3-dependent or Foxp3-independent dominant immune tolerance. J. Immunol..

