# Supplementary material for: Host lung microbiota promotes malaria-associated acute respiratory distress syndrome
Source: Nat Commun. 2022 Jun 29;13:3747. doi: 10.1038/s41467-022-31301-8 (PMC9243033; doi:10.1038/s41467-022-31301-8)
Supplement: Supplementary file 1 — Supplementary Information [file 41467_2022_31301_MOESM1_ESM.pdf]

Supplementary Information: Host lung microbiota promotes malaria-associated acute respiratory distress syndrome

Mukherjee et al

Supplementary Fig. 1

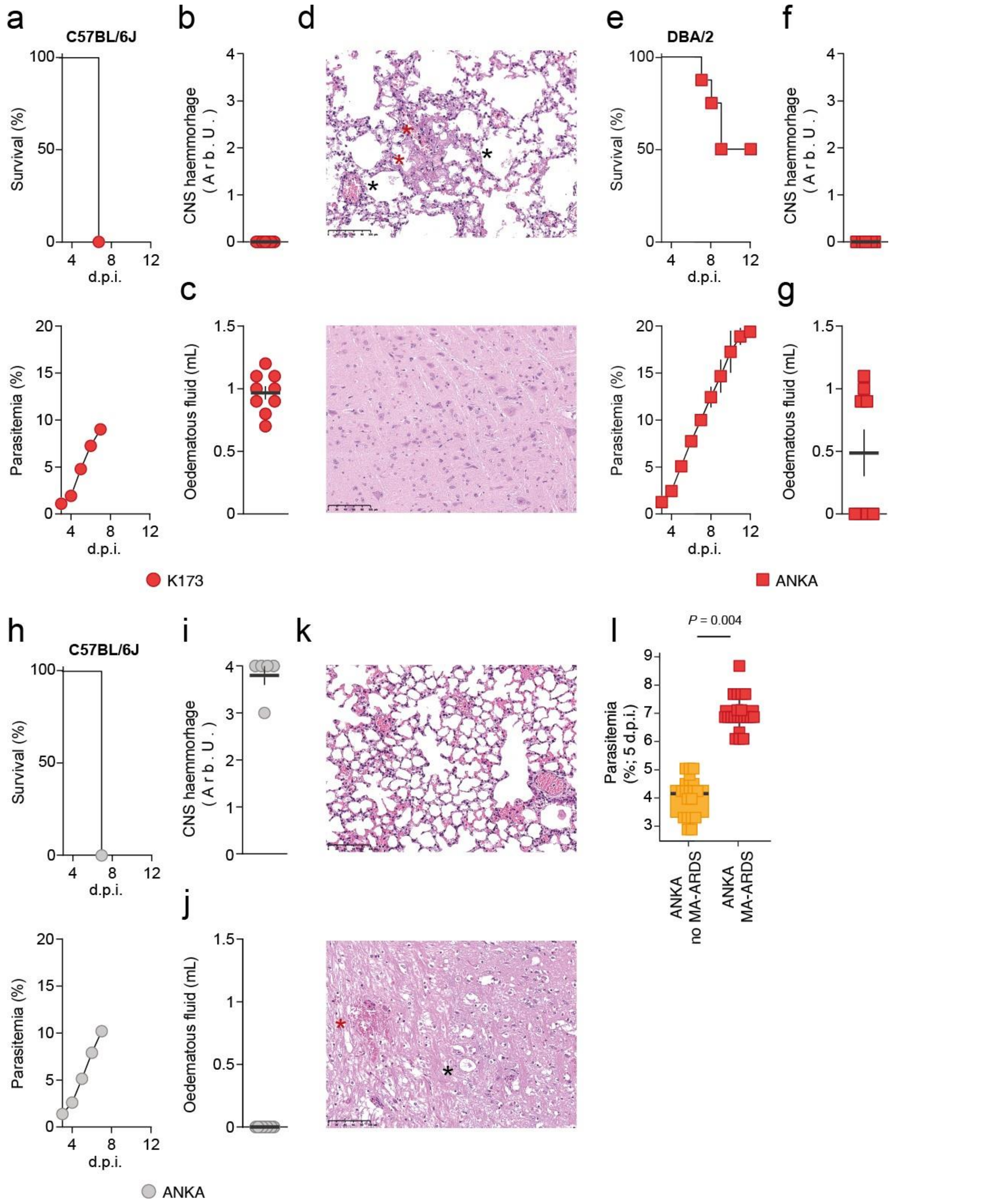

Supplementary Fig. 1 Lung microbiota dysbiosis is associated with ARDS during malaria infections. a, Survival (upper panel; Log-rank Mantel-Cox, n=5;N=1) and

parasitaemia (lower panel; linear regression n=5;N=1) of C57BL/6J mice infected with *Pb* K173. **b,** Central Nervous System (CNS) haemorrhage score (n=5;N=1) as measured by mid-brain haemorrhage severity of SPF C57BL/6J mice infected with *Pb* K173. **c,** Pulmonary oedema as measured by increased fluid content in the lungs of SPF C57BL/6J mice infected with *Pb* K173 (n=9;N=2). (Error bars are represented by mean ± s.e.m) **d,** Histological images of lung (upper panel) and CNS (lower panel) of SPF C57BL/6J mice infected with Pb K173. Alveolar oedema in the lung (black asterisk), and hyaline membranes deposition (red asterisk) were observed in the lung. No changes were observed in the brain. Scalebar = 100 µm. **e,** Survival (upper panel; Log-rank Mantel-Cox, n=5;N=1) and parasitemia (lower panel; mean ± s.e.m; linear regression, n=5;N=1) of SPF DBA/2 mice infected with *Pb* ANKA. **f,** CNS haemorrhage score (n=5;N=1) as measured by mid-brain haemorrhage severity of SPF DBA/2 mice infected with *Pb* ANKA. **g,** Pulmonary oedema as measured by increased fluid content in the lungs of SPF DBA/2 mice infected with *Pb* ANKA (n=9;N=2). (Error bars are represented by mean ± s.e.m) **h,** Survival (upper panel; Log-rank Mantel-Cox, n=10;N=1) and parasitemia (lower panel; linear regression, n=10;N=1) SPF C57BL/6J mice infected with *Pb* ANKA. **i,** CNS haemorrhage score (n=5;N=1) as measured by mid-brain haemorrhage severity of SPF C57BL/6J mice infected with *Pb* ANKA. (Error bars are represented by mean ± s.e.m) **j,** Pulmonary oedema as measured by increased fluid content in the lungs of SPF C57BL/6J mice infected with *Pb* ANKA (n=5;N=1) **k,** Histological images of lung (upper panel) and CNS (lower panel) of SPF C57BL/6J mice infected with *Pb* ANKA. No changes were observed in the lung. There were marked mid-brain hemorrhages (red asterisk) and grey matter vacuolation (black asterisk). Scale bar = 100 µm. **l,** Parasitemia (mean ± s.e.m; linear regression) following *Pb* ANKA infection of DBA/2 mice separating groups of mice that will succumb to MA-ARDS (n=20) and mice that will be protected (n=20). (Two sided Mann-Whitney test) Data is represented as floating bar plots (minimum to maximum) with line at the middle representing median. Source data are provided as a Source Data file.

Supplementary Fig. 2

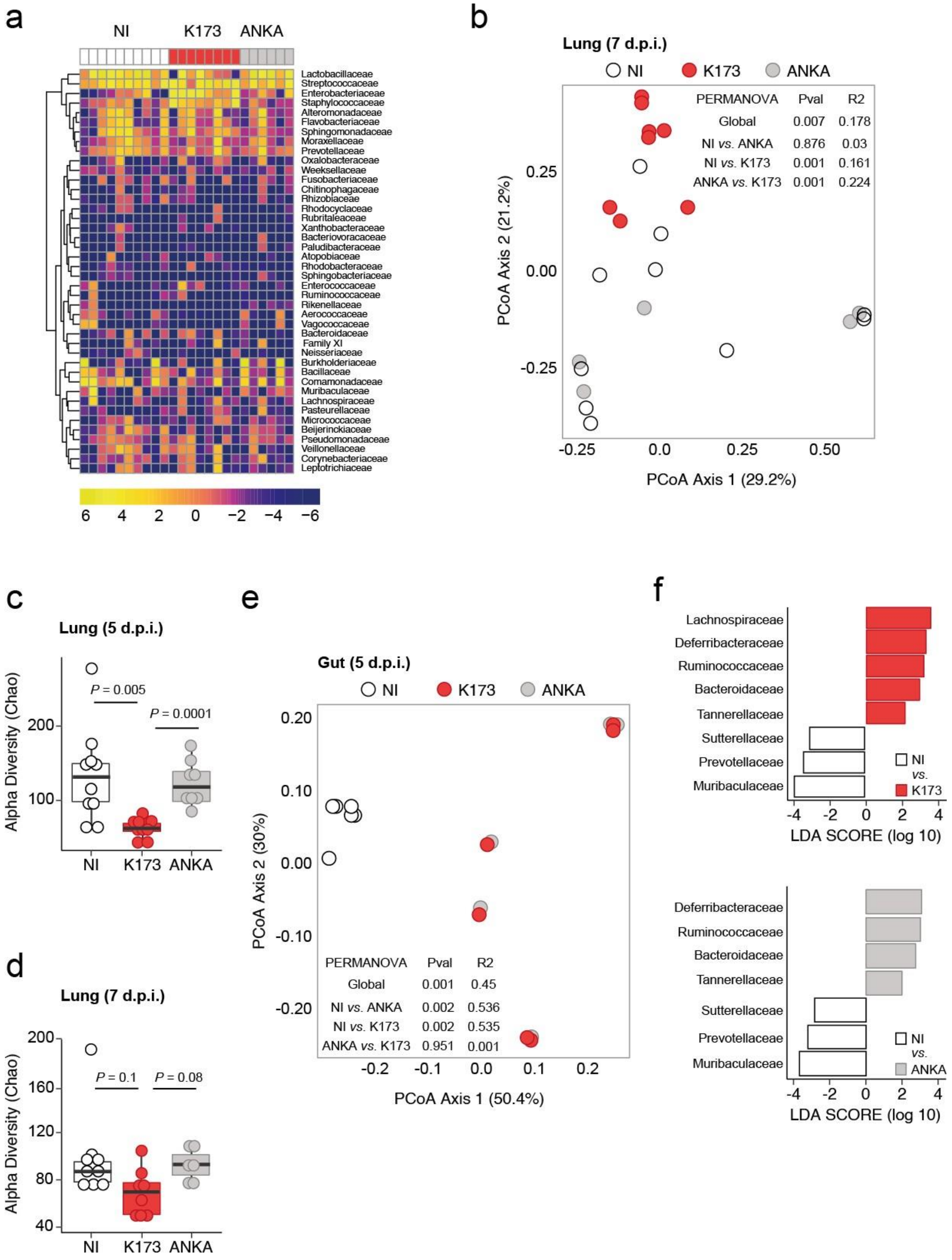

Supplementary Fig. 2 Lung microbiota dysbiosis is associated with ARDS during malaria infections. a, Heatmap of relative abundance of major microbial families in the lungs of C57BL/6J mice 7 days p.i. with *Pb* K173 (n=6;N=1) or *Pb* ANKA (n=7;N=1) and

NI controls (n=10,N=1). **b,** Beta diversity of lung microbial communities of C57BL/6J mice 5 days p.i. with *Pb* K173 (n=6;N=1) or *Pb* ANKA (n=7;N=1) and NI controls (n=10,N=1). **c,d,** Chao diversity of lung microbial composition upon infection of C57BL/6J mice **c,** after 5 days p.i. with *Pb* K173 (n=10;N=1) or *Pb* ANKA (n=8;N=1) and NI controls (n=10,N=1) and **d,** after 7 days p.i with *Pb* K173 (n=6;N=1) or *Pb* ANKA (n=7;N=1) and NI controls (n=10,N=1). For group comparision we used Kruskal-Wallis followed by two-sided Mann-Whitney post hoc analysis with Holm Correction.

e, Beta diversity of gut microbial communities of C57BL/6J mice 5 days p.i. with *Pb* K173 (n=5;N=1) or *Pb* ANKA (n=5;N=1) and NI controls (n=5,N=1). **f,** Differentially abundant bacterial families from the gut of C57BL/6J mice upon infection with *Pb* K173 (n=5;N=1 or *Pb* ANKA (n=5;N=1) and NI (n=5;N=1) controls. Bacterial families were compared using LEfSe (Linear discriminant analysis Effect Size) algorithm (Kruskal-Wallis test, $p < 0.05$, LDA score > 2.0). **c-d** Data is represented with boxplots. The lower and upper hinges represent the 1st and third quartiles . The upper whisker extends to the largest value but no further than 1.5 * inter-quartile range (IQR). The lower whisker extends to the smallest value or most 1.5 * IQR. Center hinge is the second quartile. Source data are provided as a Source Data file.

Supplementary Fig. 3

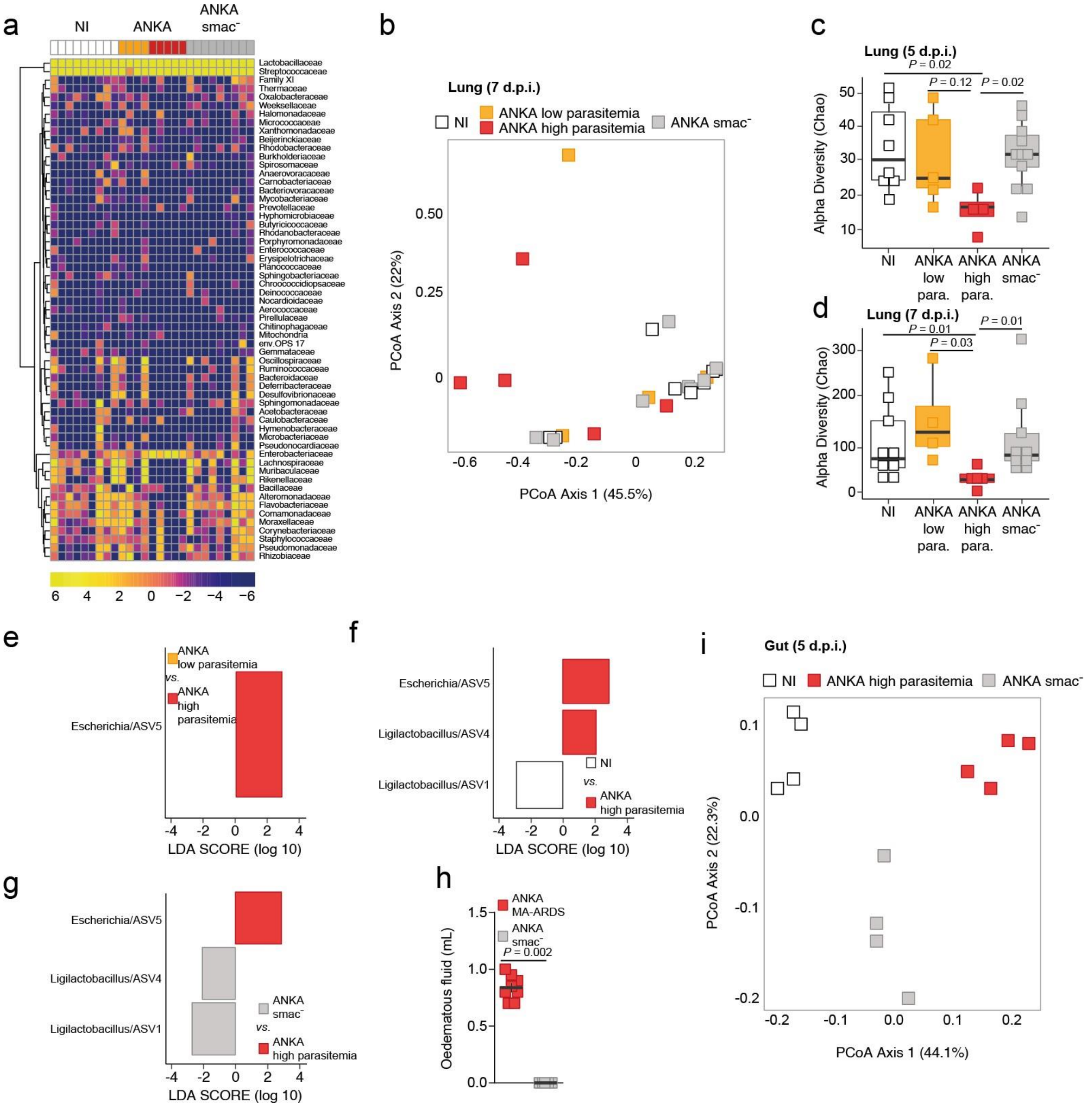

Supplementary Fig. 3 iRBCs sequestration in the lungs mediates local microbiota dysbiosis and promotes MA-ARDS. a, Heatmap of relative abundance of major microbial families in the lungs of DBA/2 mice 7 days p.i. with *Pb* ANKA high parasitemia (n=5;N=1) and *Pb* ANKA low parasitemia (n=4;N=1), *Pb* ANKA*smac-* (n=9;N=1) and NI

(n=9;N=1) controls. **b,** Beta diversity of lung microbial communities of of DBA/2 mice 7 days p.i. infection with *Pb* ANKA high parasitemia (n=5;N=1), *Pb* ANKA low parasitemia (n=4;N=1), *Pb* ANKA*smac-* (n=9;N=1) and NI (n=9;N=1) controls (statistics are provided in supplementary table 2). **c,d,** Chao diversity of lung microbial composition of DBA/2 mice upon infection **c,** with *Pb* ANKA high parasitemia (n=4;N=1), *Pb* ANKA low parasitemia (n=5;N=1), *Pb* ANKA*smac*$^{-}$ (n=9;N=1) and NI (n=7;N=1) controls 5 days p.i. and **d,** *Pb* ANKA high parasitemia (n=5;N=1), *Pb* ANKA low parasitemia (n=4;N=1), *Pb* ANKA*smac-* (n=9;N=1) and NI (n=9;N=1) controls 7 day p.i. For group comparision **we** used Kruskal-Wallis followed by two-sided Mann-Whitney post hoc analysis with Holm Correction **e-g,** Differentially abundant ASVs (using LEfSe, Linear discriminant analysis Effect Size) from lungs of DBA/2 mice comparing **e,** *Pb* ANKA high parasitemia (n=5;N=1) and *Pb* ANKA low parasitemia (n=4;N=1) at day 7 p.i. **f,** Between NI (n=9;N=1) and *Pb* high parasitemia (n=5;N=1) at day 7 **g,** between *Pb* ANKA-smac (n=9;N=1) and *Pb* ANKA high parasitemia (n=5;N=1) 7 days p.i.. **h,** Pulmonary oedema (Mann-Whitney) as measured by increased fluid content in the lungs of DBA/2 mice infected with either Pb ANKA (n=8;N=2) or Pb ANKA*smac*$^{-}$ parasites (n=8;N=2). **i,** Beta diversity analysis of gut microbial communities of DBA/2 mice 5 days p.i. with *Pb* ANKA high parasitemia (n=4;N=1), *Pb* ANKA*smac-* (n=4;N=1) and NI (n=4;N=1) controls. **c-d,** Data is represented as floating bar plots (minimum to maximum) with line at the middle representing median. Source data are provided as a Source Data file.

Supplementary Fig. 4

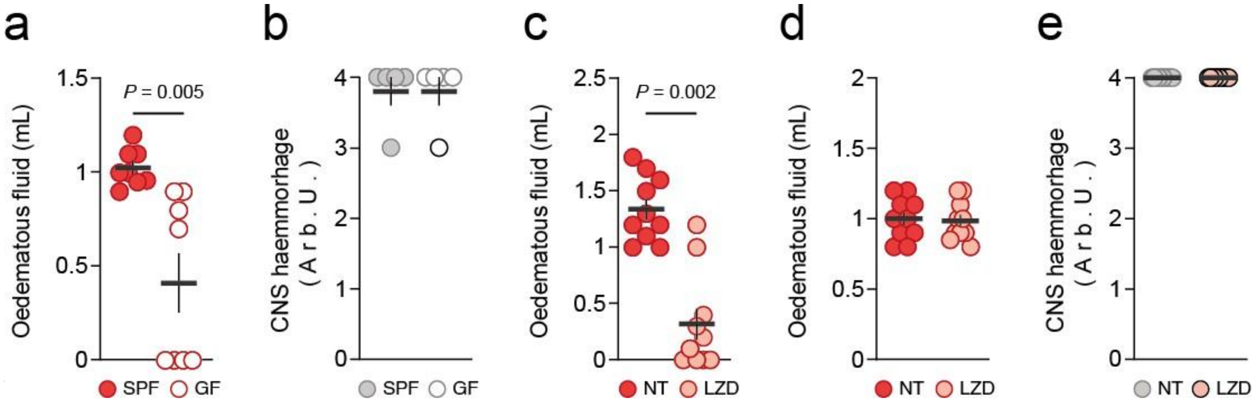

Analysis of pulmonary oedema (Two sided Mann-Whitney test) as measured by increased fluid content in the lungs of GF (n=10;N=2) or SPF (n=10;N=2) C57BL/6J mice following *Pb* K173 infection. (Error bars are represented by mean ± s.e.m) **b,** Cerebral haemorrhage (Mann-Whitney) score as measured by mid-brain haemorrhage severity following *Pb* ANKA infection of GF (n=10;N=2) or SPF (n=5;N=1) C57BL/6J mice. (Error bars are represented by mean ± s.e.m) **c,** Analysis of pulmonary oedema (Two sided Mann-Whitney test) as measured by increased fluid content in the lungs following *Pb* K173 infection of linezolid treated C57BL/6J mice (n=9;N=2) starting at 3 days p.i. compared to non-treated, *Pb* K173 infected mice (n=10;N=2)**.** (Error bars are represented by mean ± s.e.m) **d,** Analysis of pulmonary oedema (Two sided Mann-Whitney test) as measured by increased fluid content in the lungs following *Pb* K173 infection of linezolid treated C57BL/6J mice (n=10;N=2) starting at 5 days p.i. compared to non-treated, Pb K173 infected mice (n=10;N=2)**. e,** Cerebral haemorrhage score (Mann-Whitney) as measured by mid-brain haemorrhage severity following *Pb* ANKA infection of linezolid treated C57BL/6J mice (n=10;N=2) compared to non-treated, infected mice (n=6;N=2). Source data are provided as a Source Data file.

Supplementary Fig. 5

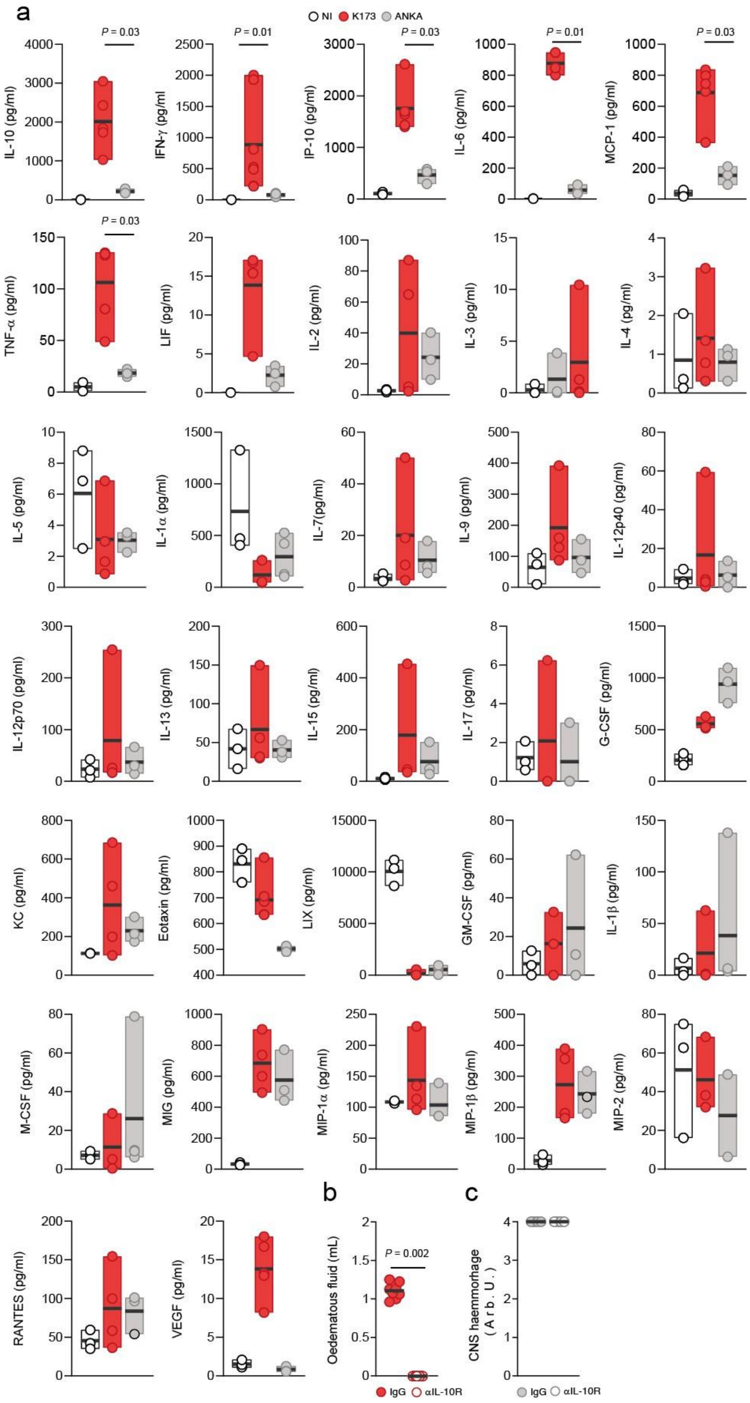

Supplementary Fig. 5 Increased IL-10 levels in the lungs mediate bacterial expansion in lung. a, 32-plex cytokine array analysis of serum of *Pb* K173 (n=5) and

Pb ANKA (n=3) infected C57BL/6J mice compared to NI controls (n=3) 5 days p.i. (Kruskal-Wallis). **b,** Pulmonary oedema (Two sided Mann-Whitney test) as measured by increased fluid content in the lungs following *Pb* K173 infection of IgG (n=10;N=2) or αIL-10R neutralizing antibody (n=10;N=2) treated C57BL/6J mice. **c,** Cerebral malaria score (Mann-Whitney) as measured by mid-brain haemorrhage severity in the brain following *Pb* ANKA infection of IgG (n=10;N=2) or αIL-10R neutralizing antibody (n=10;N=2) treated C57BL/6J mice. Source data are provided as a Source Data file.

Supplementary Fig. 6

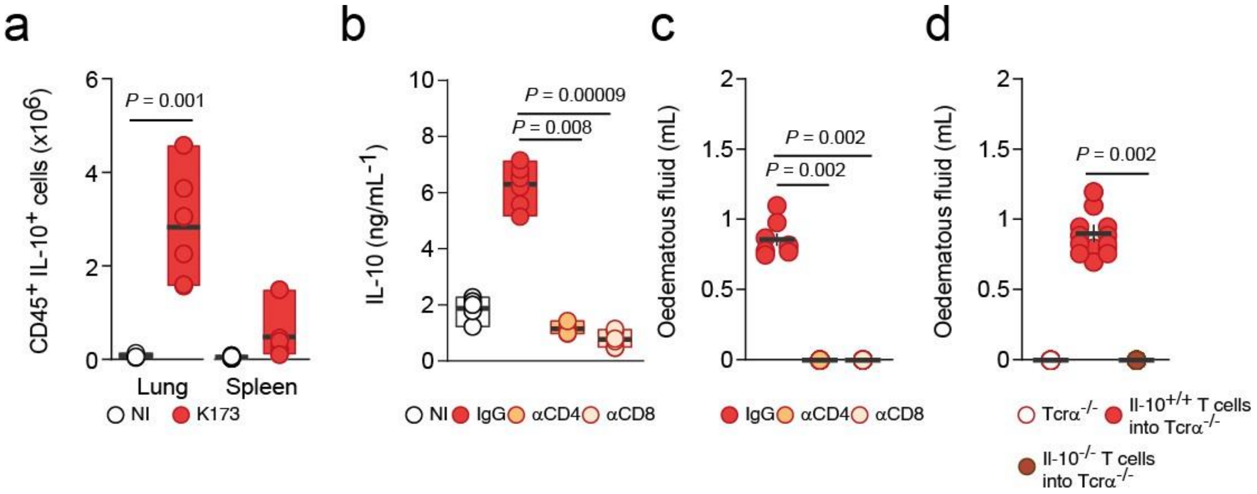

Supplementary Fig. 6 αβ T cells producing IL-10 promote altered bacterial colonization in the lung and MA-ARDS. a, Total IL-10$^+$ CD45$^+$ leukocytes in the lungs and spleen (Mann-Whitney) of C57BL/6J mice 5 days p.i. with *Pb* K173 (n=6;N=2) compared to NI (n=6;N=2) controls (Two sided Mann-Whitney test). The gating was done on live CD45$^+$ cells. **b,** Total protein levels of IL-10 (Kruskal-Wallis) in the lungs of C57BL/6J mice 5 days p.i. with *Pb* K173 treated with either αCD4 (n=6;N=2) or αCD8 (n=6;N=2) depleting antibody compared to IgG treated (n=6;N=2) and NI (n=6;N=2) controls. **c,** Pulmonary oedema (Kruskal-Wallis) as measured by increased fluid content in the lungs of IgG (n=8;N=2), αCD4 (n=8;N=2) or αCD8 (n=8;n=2) depleting antibody

treated C57BL/6J mice infected with *Pb* K173 5 days p.i.. (Error bars are represented by mean ± s.e.m) **d,** Pulmonary oedema as measured by increased fluid content in the lungs of control *Tcrα*$^{-/-}$ mice, or upon adoptive transfer of control or *Il*-10$^{-/-}$ T cells, infected with *Pb* K173 (n=12;N=2, Kruskal-Wallis). (Error bars are represented by mean ± s.e.m). Source data are provided as a Source Data file.

Supplementary Fig. 7

a

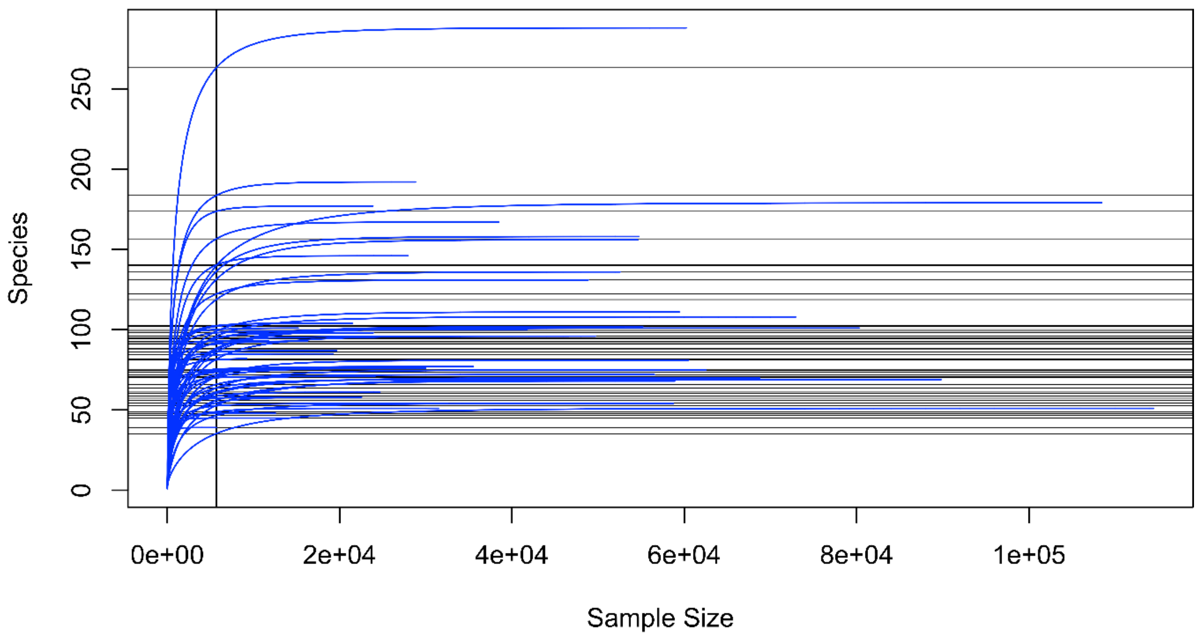

203 b

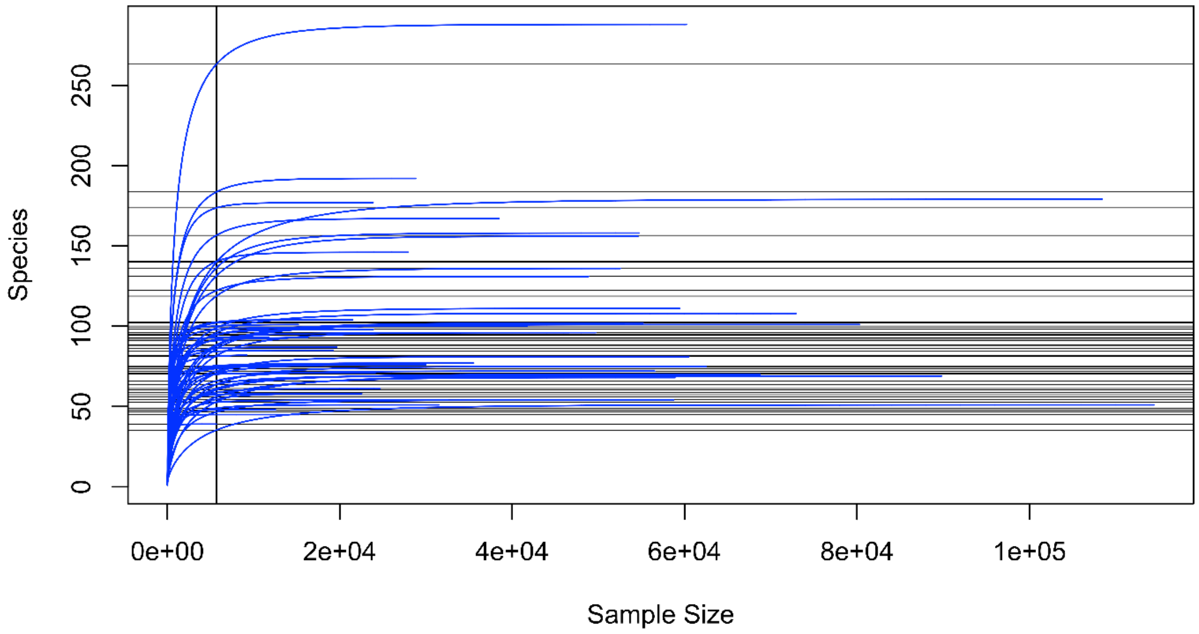

Supplementary Fig. 7 Lung microbiome quality control analysis a, Rarefaction curve showing the number of species as a function of the sample size for all C57BL/6J mice (infected and non-infected) both at day 5 and day 7 p.i. **b,** Rarefaction curve showing the number of species as a function of the sample size for all DBA/2 mice both at day 5 and day 7 p.i. Source data are provided as a Source Data file.

Supplementary Fig. 8

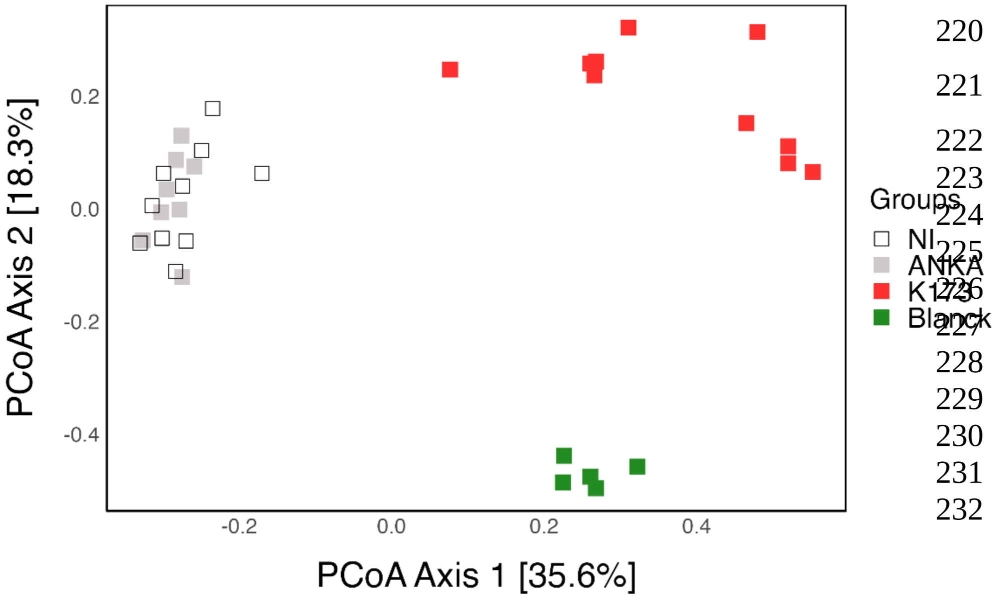

Supplementary Fig. 8 Principal coordinates analysis (PCoA), of microbial communities in samples from lungs following infection by ANKA or K173, or NI controls, as well as sequenced reads from blanks (negative controls using the same reagents for lung samples processing and sequenced in the same run). Source data are provided as a Source Data file.

Supplementary Fig. 9

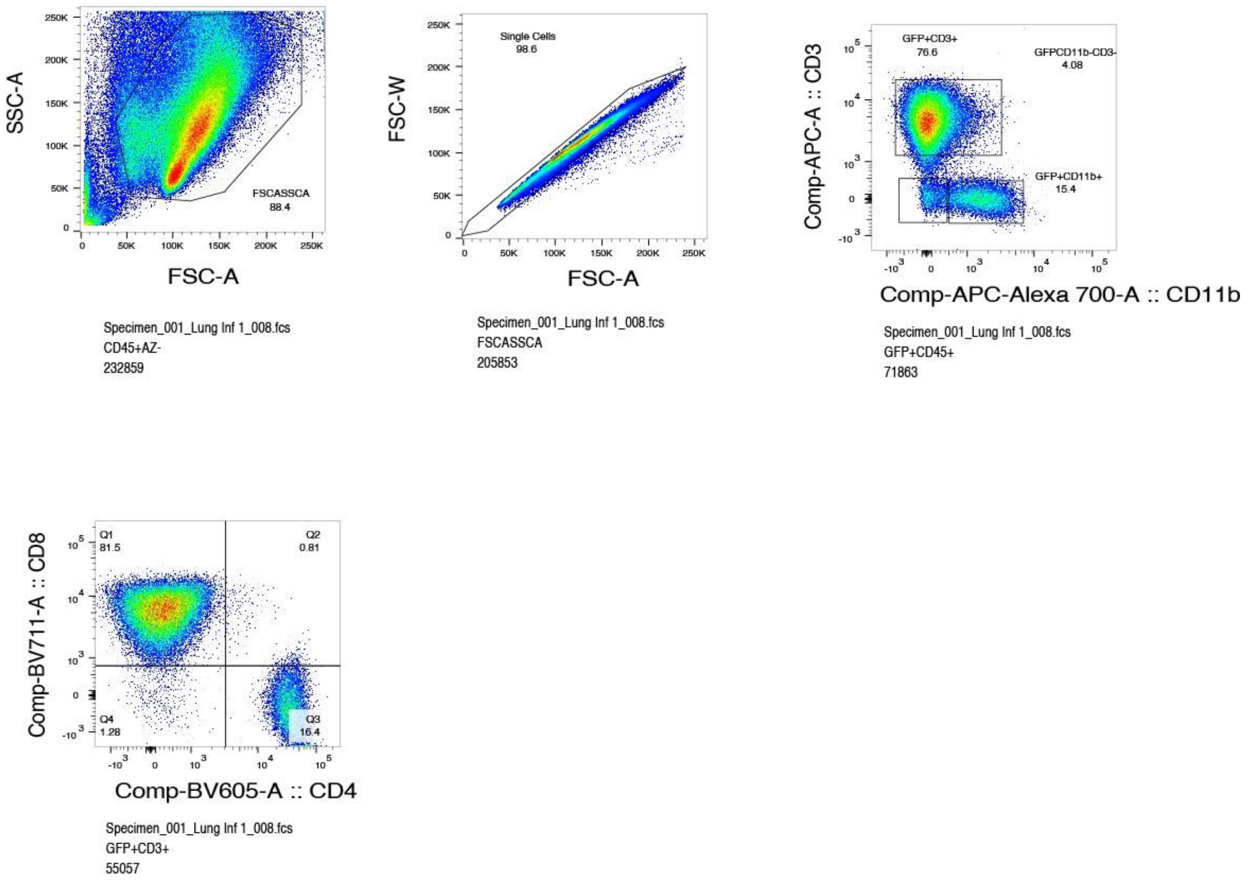

Supplementary Fig. 9 Gating strategy for flow cytometry analysis. Source data are provided as a Source Data file.

Supplementary Table 1

PERMANOVA (C57BL/6J)				
Organ	dpi	**Comparison**	**Pvalue**	**R2**
Lung	5	Global	0.001	0.485
		NI vs ANKA	0.983	0.019
		NI vs K173	0.001	0.466
		ANKA vs K173	0.001	0.474
	7	Global	0.007	0.178
		NI vs ANKA	0.876	0.03
		NI vs K173	0.001	0.161
		ANKA vs K173	0.001	0.224
Gut	5	Global	0.001	0.45
		NI vs ANKA	0.002	0.536
		NI vs K173	0.002	0.535
		ANKA vs K173	0.951	0.45

Supplementary Table 2

Permanova (DBA/2)				
Organ	dpi	**Comparison**	**Pvalue**	**R2**
Lung	5	Global	0.006	0.342
		NI vs ANKA low parasitemia	0.859	0.026
		NI vs ANKA high parasitemia	0.008	0.569
		NI vs ANKA smac-	0.673	0.026
		ANKA low parasitemia vs ANKA smac-	0.874	0.019
		ANKA high parasitemia vs ANKA smac-	0.002	0.465
		ANKA low parasitemia vs ANKA high parasitemia	0.015	0.544

	7	Global	0.006	0.342
		NI vs ANKA low parasitemia	0.321	0.091
		NI vs high parasitemia	0.012	0.305
		NI vs ANKA smac-	0.998	0.007
		ANKA low parasitemia vs ANKA smac-	0.41	0.086
		ANKA high parasitemia vs ANKA smac-	0.014	0.308
		ANKA low parasitemia vs ANKA high parasitemia	0.157	0.203
Gut	5	Global	0.001	0.63

		NI vs ANKA high parasitemia	0.03	0.63<br>9
		NI vs ANKA smac-	0.031	0.51<br>4
		ANKA high parasitemia vs ANKA smac-	0.028	0.49<br>9

Supplementary Table 3

Sample	Raw_readCount	Kraken_readCount	Fraction_kept
DBA2_A_D5_2	40656	40490	1.00
DBA2_BLANCK_D7	7366	7312	0.99
DBA2_BLANCK_D9	6674	6580	0.99
DBA2_BLANCK_D10	5300	5202	0.98
DBA2_S_D7_5	126726	123644	0.98
DBA2_NI_D7_5	156724	152594	0.97
DBA2_A_D7_5	119802	116204	0.97
DBA2_BLANCK_D12	4364	4190	0.96
DBA2_Sentinel_D7_5	162390	155318	0.96
DBA2_BLANCK_D6	15362	14664	0.95
DBA2_NI_D7_7	167594	157590	0.94
DBA2_S_D7_11	54946	51588	0.94
DBA2_S_D7_7	216344	202580	0.94
DBA2_Sentinel_D7_7	143438	133986	0.93
DBA2_A_D7_3	133756	123200	0.92
DBA2_NI_D7_3	116562	106572	0.91
DBA2_BLANCK_D11	6664	6092	0.91
B6_NI_D7_8	256552	233428	0.91
DBA2_Sentinel_D7_11	128430	116442	0.91
DBA2_S_D7_10	158572	143678	0.91
DBA2_NI_D7_2	367062	332416	0.91
DBA2_Sentinel_D7_2	253166	229100	0.90
DBA2_S_D7_2	247642	223416	0.90
DBA2_NI_D7_11	125110	112348	0.90
DBA2_Sentinel_D7_10	171482	153396	0.89
DBA2_NI_D7_10	164438	146484	0.89
DBA2_S_D7_3	230270	204718	0.89
DBA2_Sentinel_D7_3	159800	140820	0.88
B6_ANKA_D5_9	248306	218560	0.88
DBA2_NI_D7_12	93700	82406	0.88
DBA2_A_D7_8	142620	124996	0.88
DBA2_NI_D7_4	95194	83400	0.88
DBA2_Sentinel_D7_12	77484	67786	0.87

DBA2_Sentinel_D7_4	158160	138144	0.87
DBA2_BLANCK_D5	31850	27796	0.87
B6_A_D7_1	188672	164612	0.87
B6_ANKA_D5_6	346460	301482	0.87
DBA2_S_D7_4	181082	156374	0.86
DBA2_NI_D7_9	160582	138452	0.86
DBA2_Sentinel_D7_9	169398	145952	0.86
DBA2_S_D7_9	210508	179216	0.85
DBA2_S_D7_12	74906	63538	0.85
B6_BLANCK_C12	148834	124550	0.84
DBA2_BLANCK_D8	207560	170036	0.82
B6_BLANCK_C9	81122	66302	0.82
B6_BLANCK_F4	35202	28626	0.81
B6_BLANCK_F3	112968	91614	0.81
B6_ANKA_D5_8	260022	210816	0.81
B6_BLANCK_G12	680912	544836	0.80
DBA2_NI_D5_5	158186	125338	0.79
B6_A_D7_7	307570	242788	0.79
B6_ANKA_D5_7	323850	251774	0.78
B6_BLANCK_C7	537326	414212	0.77
DBA2_S_D5_1	139410	106568	0.76
DBA2_Sentinel_D7_8	72670	54872	0.76
DBA2_S_D7_8	64786	48588	0.75
DBA2_A_D7_10	31402	23504	0.75
DBA2_A_D5_5	109204	81522	0.75
B6_BLANCK_C8	152876	113834	0.74
DBA2_NI_D7_8	62406	46332	0.74
B6_ANKA_D5_4	252692	184590	0.73
B6_K_D5_7	424488	308300	0.73
B6_BLANCK_C11	536702	388998	0.72
DBA2_A_D7_9	124336	88864	0.71
DBA2_A_D7_11	294512	210110	0.71
B6_BLANCK_G11	448134	319476	0.71
DBA2_A_D5_4	196358	138672	0.71
B6_A_D7_2	145264	97368	0.67
B6_BLANCK_C10	672026	449012	0.67
B6_ANKA_D5_10	333162	221068	0.66
DBA2_A_D7_2	130076	85012	0.65
DBA2_Sentinel_D7_6	75704	49268	0.65

DBA2_NI_D7_6	88504	56506	0.64
DBA2_S_D7_6	97340	60964	0.63
B6_NI_D5_10	364524	225268	0.62
DBA2_S_D5_9	71998	43682	0.61
B6_NI_D7_1	298036	180640	0.61
B6_K_D5_8	364242	219788	0.60
DBA2_Sentinel_D5_8	39376	23514	0.60
B6_NI_D7_10	257984	152980	0.59
DBA2_NI_D5_9	82036	48638	0.59
DBA2_A_D7_6	99108	57278	0.58
DBA2_A_D7_7	232510	134038	0.58
DBA2_S_D5_4	50190	28836	0.57
DBA2_A_D7_4	186198	106744	0.57
B6_ANKA_D5_3	493360	279796	0.57
DBA2_NI_D5_8	46624	26176	0.56
DBA2_NI_D5_10	20672	11544	0.56
B6_A_D7_8	263266	146606	0.56
DBA2_A_D5_10	108456	59700	0.55
DBA2_Sentinel_D5_4	42324	22992	0.54
B6_K_D5_1	302152	160774	0.53
B6_K_D5_3	306314	162130	0.53
DBA2_A_D5_9	102236	53738	0.53
B6_K_D5_4	240964	125238	0.52
B6_NI_D5_5	347164	179316	0.52
DBA2_S_D5_8	86920	44848	0.52
B6_K_D5_9	307520	156552	0.51
B6_K_D7_2	399442	202492	0.51
B6_ANKA_D5_2	344254	172254	0.50
B6_ANKA_D5_5	334668	167314	0.50
DBA2_NI_D5_4	68558	34266	0.50
B6_NI_D5_8	378266	186770	0.49
B6_NI_D7_2	345520	164522	0.48
B6_NI_D7_6	246132	113434	0.46
B6_K_D5_6	369804	169620	0.46
B6_A_D7_5	491890	225304	0.46
B6_K_D5_10	375674	171528	0.46
B6_NI_D5_1	421142	191376	0.45
B6_K_D7_4	315874	142264	0.45
B6_A_D7_4	209534	89358	0.43

B6_A_D7_6	208276	88056	0.42
B6_K_D7_5	220422	93124	0.42
DBA2_Sentinel_D5_9	62230	25178	0.40
DBA2_A_D5_1	231098	91716	0.40
B6_NI_D7_3	231500	91816	0.40
DBA2_S_D5_6	34640	13330	0.38
B6_K_D5_5	561402	206582	0.37
B6_NI_D5_3	499492	178976	0.36
DBA2_S_D5_10	55354	19346	0.35
DBA2_Sentinel_D5_6	18420	6388	0.35
B6_NI_D5_2	428222	148282	0.35
B6_NI_D7_9	244148	83880	0.34
B6_ANKA_D5_1	7500	2516	0.34
DBA2_S_D5_2	26570	8762	0.33
B6_NI_D7_7	416218	135396	0.33
DBA2_NI_D5_7	178130	55862	0.31
B6_NI_D5_4	490884	151940	0.31
DBA2_NI_D5_2	12470	3708	0.30
DBA2_A_D5_7	51918	15154	0.29
DBA2_NI_D5_6	33812	9718	0.29
B6_K_D7_1	328184	94226	0.29
B6_NI_D7_4	399860	113918	0.28
DBA2_Sentinel_D5_2	13244	3716	0.28
DBA2_S_D5_5	342970	92682	0.27
B6_A_D7_3	556186	148442	0.27
B6_K_D5_2	287038	76484	0.27
DBA2_S_D5_3	124208	30578	0.25
B6_NI_D5_7	479016	115578	0.24
B6_NI_D5_9	483180	116512	0.24
DBA2_Sentinel_D5_1	383332	90814	0.24
B6_K_D7_6	246496	56984	0.23
DBA2_Sentinel_D7_1	385588	89114	0.23
DBA2_S_D5_7	115700	26706	0.23
B6_NI_D7_5	272754	62184	0.23
DBA2_A_D7_1	403044	90914	0.23
B6_K_D7_8	387346	85674	0.22
DBA2_Sentinel_D5_5	620148	134112	0.22
DBA2_A_D7_12	202856	40824	0.20
DBA2_S_D7_1	451066	86278	0.19

B6_K_D7_7	436204	75608	0.17
DBA2_Sentinel_D5_10	36466	6284	0.17
B6_NI_D5_6	498498	80232	0.16
DBA2_A_D5_8	191604	25316	0.13
DBA2_NI_D7_1	650930	84244	0.13
B6_K_D7_3	401886	51764	0.13
DBA2_NI_D5_3	341154	41114	0.12
DBA2_Sentinel_D5_3	576102	40198	0.07
DBA2_Sentinel_D5_7	348388	24104	0.07
DBA2_A_D5_3	280792	12744	0.05
