# Supplementary material for: Host lung microbiota promotes malaria-associated acute respiratory distress syndrome
Source: Nat Commun. 2022 Jun 29;13:3747. doi: 10.1038/s41467-022-31301-8 (PMC9243033; doi:10.1038/s41467-022-31301-8)
Supplement: Supplementary file 2 — Reporting Summary [file 41467_2022_31301_MOESM2_ESM.pdf]

nature portfolio

Corresponding author(s): Maria Manuel Mota

Last updated by author(s): June 8, 2022

Reporting Summary

Nature Portfolio wishes to improve the reproducibility of the work that we publish. This form provides structure for consistency and transparency in reporting. For further information on Nature Portfolio policies, see our Editorial Policies and the Editorial Policy Checklist.

Statistics

For all statistical analyses, confirm that the following items are present in the figure legend, table legend, main text, or Methods section.

n/a	Confirmed	
☐	☒	The exact sample size (*n*) for each experimental group/condition, given as a discrete number and unit of measurement
☐	☒	A statement on whether measurements were taken from distinct samples or whether the same sample was measured repeatedly
☐	☒	The statistical test(s) used AND whether they are one- or two-sided<br>*Only common tests should be described solely by name; describe more complex techniques in the Methods section.*
☐	☒	A description of all covariates tested
☐	☒	A description of any assumptions or corrections, such as tests of normality and adjustment for multiple comparisons
☐	☒	A full description of the statistical parameters including central tendency (e.g. means) or other basic estimates (e.g. regression coefficient) AND variation (e.g. standard deviation) or associated estimates of uncertainty (e.g. confidence intervals)
☐	☒	For null hypothesis testing, the test statistic (e.g. *F*, *t*, *r*) with confidence intervals, effect sizes, degrees of freedom and *P* value noted<br>*Give P values as exact values whenever suitable.*
☒	☐	For Bayesian analysis, information on the choice of priors and Markov chain Monte Carlo settings
☒	☐	For hierarchical and complex designs, identification of the appropriate level for tests and full reporting of outcomes
☒	☐	Estimates of effect sizes (e.g. Cohen's *d*, Pearson's *r*), indicating how they were calculated

Our web collection on statistics for biologists contains articles on many of the points above.

Software and code

Policy information about availability of computer code

Data collection	Flow Cytometry: data was collected on LSRFortessa-X20 analyzers using FACSDiva Software Versions 8.0 (LSRFortessa-X20) (BD Biosciences).<br>Total cell number: data was collected in a Accuri C6 Plus analyzer (BD Biosciences) using Accuri C6 software 1.0.264.21.
Data analysis	Flow Cytometry: Flow Cytometry Standard (FCS) 3.0 files were analyzed using FlowJo-V10 software (Tree Star Inc.).<br>GraphPad (Prism) 5.0 software was also used for statistical analysis.<br>Microbiome analysis:<br>1. Contaminating sequences were identified and removed using decontam (v1.12.0) based on identities of ASVs from blanks in the lung samples.We did not perform this for the gut samples.<br>2. Each lung sample was cleaned of Plasmodium parasite and host sequences using Kraken2. To do this, we first created a Kraken2 index consisting of the Bacteria and Archaea libraries plus the mouse genome (GRCm39) and the genomes of Plasmodium berghei ANKA (GCA_900002375.2) and K173 (GCA_900044334.1), all of which were downloaded from NCBI. The reads of each sample were then mapped against this database with Kraken2 and the script extract_kraken_reads.py (from KrakenTools; https://github.com/jenniferlu717/KrakenTools) was used to filter reads in order to keep only those mapping to Bacteria or Archaea for further analysis. Since gut samples do not have so much host contaminants this step was not done.<br>3. All analysis of both lung and gut microbiota composition were performed on R (v4.0.2). For data manipulation, we used the packages dplyr (v 1.0.5) and tidyverse (v1.3.0).<br>4. Rarefication of the data was done using rrarefy (vegan v2.5.7) for the lung samples.This was not done for the gut samples. We compared the microbiome between experimental groups for both lung and gut samples with Permutational Multivariate ANalysis Of Variance (PERMANOVA) using the function adonis (vegan v2.5.7) with 999 permutations. Dissimilarity distances (Bray-Curtis) were calculated using vegdist (vegan v2.5.7) then reduced to principal coordinates using betadisper (vegan v2.5.7) and finally plotted using ggplot2 (v3.3.3).<br>5. To find the differentially abundant ASVs for both lung and gut samples, we transformed our data into a SummarizedExperiment object using SummarizedExperiment (v1.20.0), then we ran Linear discriminant analysis Effect Size (LEfSe) algorithm (Kruskal-Wallis test, $p < 0.05$, LDA score > 2.0) using lefser (v1.0.0).

6. The heatmaps for the lung samples were generated using pheatmap (pheatmap v1.0.12). Diversity calculations for the lung samples were done at ASV levels using diversity function (vegan v2.5.7) for Shannon index and Chao1 (fossil v 0.4.0). There was no heatmap or diversity calculations for gut samples in this manuscript.

Figures in the manuscript:
All the figures in the manuscript were generated using Adobe Illustrator (version CS6).

For manuscripts utilizing custom algorithms or software that are central to the research but not yet described in published literature, software must be made available to editors and reviewers. We strongly encourage code deposition in a community repository (e.g. GitHub). See the Nature Portfolio guidelines for submitting code & software for further information.

Data

Policy information about availability of data

All manuscripts must include a data availability statement. This statement should provide the following information, where applicable:

- Accession codes, unique identifiers, or web links for publicly available datasets
- A description of any restrictions on data availability
- For clinical datasets or third party data, please ensure that the statement adheres to our policy

Data generated for this manuscript is available. 16S rRNA amplicon sequencing data are deposited in the SRA database with the BioProject accession number PRJNA746689. The link for the sequencing data is https://www.ncbi.nlm.nih.gov/sra/PRJNA746689. Each lung sample was cleaned of Plasmodium parasite and host sequences using Kraken2. To do this, we first created a Kraken2 index consisting of the Bacteria and Archaea libraries plus the mouse genome (GRCm39) and the genomes of Plasmodium berghei ANKA (GCA_900002375.2) and K173 (GCA_900044334.1), all of which were downloaded from NCBI. The reads of each sample were then mapped against this database with Kraken2 and the script extract_kraken_reads.py (from KrakenTools; https://github.com/jenniferlu717/KrakenTools) was used to filter reads in order to keep only those mapping to Bacteria or Archaea for further analysis (Supplementary Table 3).

Field-specific reporting

Please select the one below that is the best fit for your research. If you are not sure, read the appropriate sections before making your selection.

- [x] Life sciences
- [ ] Behavioural & social sciences
- [ ] Ecological, evolutionary & environmental sciences

For a reference copy of the document with all sections, see nature.com/documents/nr-reporting-summary-flat.pdf

Life sciences study design

All studies must disclose on these points even when the disclosure is negative.

Sample size	Most experiments were performed with 3 independent replicates. Sample sizes were chosen on the basis of historical data with 9-10 animals per group (Mancio-Silva et al Nature, 2018; no statistical methods were used to predetermine sample size. Biological replicates (n) refering to the number of mice, pooled from experiments performed independently (N), are indicated in the figure legends.
Data exclusions	No data was excluded in this study.
Replication	All experiments were performed more than once on an independent manner (biological replicates). The experimental findings were reliably reproduced as validated by at least three independent experiments
Randomization	Animals were allocated randomly into experimental groups.
Blinding	The investigators were not blinded. Blinding was not relevant to our study since most results depend on machine acquisition (except for following parasitaemia's that were performed on arbitrarily chosen fields of view in the microscope of Giemsa stained smears) followed by human analysis reviewed by an independent investigator. Blinding was not required to group allocation during data collection and/or analysis since this is the standard approach for the experiments described in the manuscript, as there were no subjective measurements.

Reporting for specific materials, systems and methods

We require information from authors about some types of materials, experimental systems and methods used in many studies. Here, indicate whether each material, system or method listed is relevant to your study. If you are not sure if a list item applies to your research, read the appropriate section before selecting a response.

Materials & experimental systems

n/a	Involved in the study
☐	☒ Antibodies
☐	☐ Eukaryotic cell lines
☐	☐ Palaeontology and archaeology
☐	☒ Animals and other organisms
☐	☐ Human research participants
☐	☐ Clinical data
☐	☐ Dual use research of concern

Methods

n/a	Involved in the study
☐	☐ ChIP-seq
☐	☒ Flow cytometry
☐	☐ MRI-based neuroimaging

Antibodies

Antibodies used

α-IL-10R rat monoclonal antibody (clone 1B1.2), anti-CD4 mouse (clone YTA 3.1) and anti-CD8 mouse (clone YTS 156/169) were kindly provided by Dr. Luis Graça Lab. IL-10 signalling was blocked by injecting, by intraperitoneal route, 100µg of α-IL-10R monoclonal antibody at days 0, 2 and 4 post infection. Control groups were injected with rat immunoglobulin in parallel (Rat IgG2b Biolegend, catalog number: 400602). For in vivo CD4+ and CD8+ T cell depletion experiments, mice were injected intraperitoneally with 50µg of each antibody individually or the corresponding isotype control (rat IgG2b Biocell, catalogue number: BE0117) at days 0 and 3 post infection.

For direct multi-colour flow cytometry (LRS Fortessa X-20; BD Bioscience), cells were incubated for 30mins at 4°C with the antibodies mentioned below. Live/dead cell discrimination was performed by staining with the Zombie Aqua Fixable Viability Kit (BioLegend, catalogue number: 423101). I To block Fc receptors, a purified anti-mouse CD16/CD32 (eBioscience, catalogue number: 14-0161-86, 1:100) was used.

CD19 APCCy7 (clone 6D5, Biolegend, catalogue number: 115530, 1:300), Ly6G PerCPCy5.5 (clone 1A8, Biolegend, catalogue number: 127616, 1:300), CD45 PEDazzle (clone 30-F11, Biolegend, catalogue number: 103146, 1:400), F4/80PECy7 (clone BM8, eBioscience, catalog number:254801-82, 1:200), CD3 APC (clone 145-2c11, Biolegend, catalogue number: 100312, 1:100), CD11b Alexa700 (clone M170, Biolegend, catalogue number: 101222, 1:400), TCγδV421 (clone GL3, Biolegend, catalog number: 118120, 1:100), CD4 BV605 (clone RM4-5, Biolegend, catalogue number: 100548, 1:300), CD8 BV711 (clone 53-6.7, Biolegend, catalogue number: 100748, 1:300) and NK1.1 PE (clone PK136, Biolegend, catalogue number: 557391, 1:300).

Validation

Flow Cytometry: Fluorochrome-conjugated monoclonal antibodies were purchased from commercial vendors. Any information on the validation performed by the manufacturer can be obtained from the manufacturer's website.

Eukaryotic cell lines

Policy information about cell lines

Cell line source(s)

State the source of each cell line used.

Authentication

Describe the authentication procedures for each cell line used OR declare that none of the cell lines used were authenticated.

Mycoplasma contamination

Confirm that all cell lines tested negative for mycoplasma contamination OR describe the results of the testing for mycoplasma contamination OR declare that the cell lines were not tested for mycoplasma contamination.

Commonly misidentified lines
(See ICLAC register)

Name any commonly misidentified cell lines used in the study and provide a rationale for their use.

Palaeontology and Archaeology

Specimen provenance

Provide provenance information for specimens and describe permits that were obtained for the work (including the name of the issuing authority, the date of issue, and any identifying information). Permits should encompass collection and, where applicable, export.

Specimen deposition

Indicate where the specimens have been deposited to permit free access by other researchers.

Dating methods

If new dates are provided, describe how they were obtained (e.g. collection, storage, sample pretreatment and measurement), where they were obtained (i.e. lab name), the calibration program and the protocol for quality assurance OR state that no new dates are provided.

☐ Tick this box to confirm that the raw and calibrated dates are available in the paper or in Supplementary Information.

Ethics oversight

Identify the organization(s) that approved or provided guidance on the study protocol, OR state that no ethical approval or guidance was required and explain why not.

Note that full information on the approval of the study protocol must also be provided in the manuscript.

Animals and other organisms

Policy information about studies involving animals; ARRIVE guidelines recommended for reporting animal research

Laboratory animals	Male C57BL/6J and DBA/2 mice were purchased from Charles River breeding laboratories and housed in the animal facilities at Instituto de Medicina Molecular João Lobo Antunes (iMM-JLA) and Instituto Gulbenkian de Ciência (IGC) in specific pathogen-free (SPF) conditions. Male germ-free mice were generated at the Germ Free breeding facility at IGC and housed in gnotobiotic conditions. Male C57BL/6J Vert-X IL-10GFP reporter mice were obtained from the animal house at the Francis Crick Institute in London, United Kingdom and, like the male TCRα-deficient mice (Jackson laboratories Stock No:004364), housed at the SPF facility of iMM-JLA. All animals used in this study was between 4-6 weeks in age. Animals were housed under the following conditions with 14h Light:10h Dark cycle, temperature 20-240C and relative humidity of 55±10%.
Wild animals	The study did not involve wild animals
Field-collected samples	The study did not involve samples collected from the field
Ethics oversight	Animal experiments were performed according to EU regulations and approved by the Órgão Responsável pelo Bem-estar Animal (ORBEA) of Instituto de Medicina Molecular and by the Direcção-Geral de Alimentação e Veterinária (Portugal),

Note that full information on the approval of the study protocol must also be provided in the manuscript.

Human research participants

Policy information about studies involving human research participants

Population characteristics	*Describe the covariate-relevant population characteristics of the human research participants (e.g. age, gender, genotypic information, past and current diagnosis and treatment categories). If you filled out the behavioural & social sciences study design questions and have nothing to add here, write "See above."*
Recruitment	*Describe how participants were recruited. Outline any potential self-selection bias or other biases that may be present and how these are likely to impact results.*
Ethics oversight	*Identify the organization(s) that approved the study protocol.*

Note that full information on the approval of the study protocol must also be provided in the manuscript.

Clinical data

Policy information about clinical studies

All manuscripts should comply with the ICMJEguidelines for publication of clinical research and a completedCONSORT checklist must be included with all submissions.

Clinical trial registration	*Provide the trial registration number from ClinicalTrials.gov or an equivalent agency.*
Study protocol	*Note where the full trial protocol can be accessed OR if not available, explain why.*
Data collection	*Describe the settings and locales of data collection, noting the time periods of recruitment and data collection.*
Outcomes	*Describe how you pre-defined primary and secondary outcome measures and how you assessed these measures.*

Dual use research of concern

Policy information about dual use research of concern

Hazards

Could the accidental, deliberate or reckless misuse of agents or technologies generated in the work, or the application of information presented in the manuscript, pose a threat to:

No	Yes	
☐	☐	Public health
☐	☐	National security
☐	☐	Crops and/or livestock
☐	☐	Ecosystems
☐	☐	Any other significant area

Experiments of concern

Does the work involve any of these experiments of concern:

No	Yes	
☐	☐	Demonstrate how to render a vaccine ineffective
☐	☐	Confer resistance to therapeutically useful antibiotics or antiviral agents
☐	☐	Enhance the virulence of a pathogen or render a nonpathogen virulent
☐	☐	Increase transmissibility of a pathogen
☐	☐	Alter the host range of a pathogen
☐	☐	Enable evasion of diagnostic/detection modalities
☐	☐	Enable the weaponization of a biological agent or toxin
☐	☐	Any other potentially harmful combination of experiments and agents

ChIP-seq

Data deposition

☐ Confirm that both raw and final processed data have been deposited in a public database such as GEO.

☐ Confirm that you have deposited or provided access to graph files (e.g. BED files) for the called peaks.

Data access links<br>*May remain private before publication.*	*For "Initial submission" or "Revised version" documents, provide reviewer access links. For your "Final submission" document, provide a link to the deposited data.*
Files in database submission	*Provide a list of all files available in the database submission.*
Genome browser session<br>(e.g. UCSC)	*Provide a link to an anonymized genome browser session for "Initial submission" and "Revised version" documents only, to enable peer review. Write "no longer applicable" for "Final submission" documents.*

Methodology

Replicates	*Describe the experimental replicates, specifying number, type and replicate agreement.*
Sequencing depth	*Describe the sequencing depth for each experiment, providing the total number of reads, uniquely mapped reads, length of reads and whether they were paired- or single-end.*
Antibodies	*Describe the antibodies used for the ChIP-seq experiments; as applicable, provide supplier name, catalog number, clone name, and lot number.*
Peak calling parameters	*Specify the command line program and parameters used for read mapping and peak calling, including the ChIP, control and index files used.*
Data quality	*Describe the methods used to ensure data quality in full detail, including how many peaks are at FDR 5% and above 5-fold enrichment.*
Software	*Describe the software used to collect and analyze the ChIP-seq data. For custom code that has been deposited into a community repository, provide accession details.*

Flow Cytometry

Plots

Confirm that:

☒ The axis labels state the marker and fluorochrome used (e.g. CD4-FITC).

☒ The axis scales are clearly visible. Include numbers along axes only for bottom left plot of group (a 'group' is an analysis of identical markers).

☒ All plots are contour plots with outliers or pseudocolor plots.

☒ A numerical value for number of cells or percentage (with statistics) is provided.

Methodology

Sample preparation	Lungs were harvested from C57BL/6J Vert-X IL-10GFP reporter mice and washed with sterile 1x PBS and placed in Petri dishes with RPMI 1640 medium (3ml) (Sigma-Aldrich, catalogue number: C5138). Whole lung was minced with forceps and scissors to 1mm sized pieces and incubated with 3ml of digestion medium (2mg of Collagenase type IV, 3ml of RPMI 1640 medium with Type IV DNase I (final concentration: 25units/ml, Sigma-Aldrich, catalogue number: C5138) at 37°C under agitation (200rpm) conditions for 45mins. Following that, tissue digestion was stopped by adding 1 ml of heat-inactivated FBS (Life

Technologies, catalogue number: 10437-028). The cells were then dispersed with a 10ml syringe (BD Biosciences, catalogue number: 301604) fitted with an 18gauge needle (10times) (BD Biosciences, catalogue number: 305180) and filtered using a cell strainer (100µm, Corning, catalogue number: 352360). The cells were then centrifuged (Centrifuge 5810 Eppendorf) at 10 °C and 300 x g for 5 min. The supernatant was discarded and 1 ml of red blood cell lysis buffer (0.144M NH_4Cl, 0.0169 M TRIS base, pH 7.4) was added and incubated at room temperature for 1min. The reaction was stopped by adding 10ml of 1xPBS with 10% heat inactivated FBS. Finally, the cells were centrifuge at 10°C and 300 x g for 5mins and the pellet was resuspended in FACS buffer (PBS + 2%FCS) for further staining.

Instrument

BD LSRFortessa™ X-20

Software

Flow cytometry data was collected using FACSDiva Software Versions 8.0 (LSRFortessa-X20) and analyzed using FlowJo-V10 software (Tree Star Inc.).

Cell population abundance

No cell sorting was performed in the present study.

Gating strategy

For all analysis, cells were selected on the basis of physical parameters (FSC-A vs SSC-A) and doublets were excluded (FSC-A vs FSC-W). CD8+ and CD4+ T lymphocytes infiltrating the lung was gated on live CD45+CD3+ cells.

☒ Tick this box to confirm that a figure exemplifying the gating strategy is provided in the Supplementary Information.

Magnetic resonance imaging

Experimental design

Design type

Indicate task or resting state; event-related or block design.

Design specifications

Specify the number of blocks, trials or experimental units per session and/or subject, and specify the length of each trial or block (if trials are blocked) and interval between trials.

Behavioral performance measures

State number and/or type of variables recorded (e.g. correct button press, response time) and what statistics were used to establish that the subjects were performing the task as expected (e.g. mean, range, and/or standard deviation across subjects).

Acquisition

Imaging type(s)

Specify: functional, structural, diffusion, perfusion.

Field strength

Specify in Tesla

Sequence & imaging parameters

Specify the pulse sequence type (gradient echo, spin echo, etc.), imaging type (EPI, spiral, etc.), field of view, matrix size, slice thickness, orientation and TE/TR/flip angle.

Area of acquisition

State whether a whole brain scan was used OR define the area of acquisition, describing how the region was determined.

Diffusion MRI ☐ Used ☐ Not used

Preprocessing

Preprocessing software

Provide detail on software version and revision number and on specific parameters (model/functions, brain extraction, segmentation, smoothing kernel size, etc.).

Normalization

If data were normalized/standardized, describe the approach(es): specify linear or non-linear and define image types used for transformation OR indicate that data were not normalized and explain rationale for lack of normalization.

Normalization template

Describe the template used for normalization/transformation, specifying subject space or group standardized space (e.g. original Talairach, MNI305, ICBM152) OR indicate that the data were not normalized.

Noise and artifact removal

Describe your procedure(s) for artifact and structured noise removal, specifying motion parameters, tissue signals and physiological signals (heart rate, respiration).

Volume censoring

Define your software and/or method and criteria for volume censoring, and state the extent of such censoring.

Statistical modeling & inference

Model type and settings

Specify type (mass univariate, multivariate, RSA, predictive, etc.) and describe essential details of the model at the first and second levels (e.g. fixed, random or mixed effects; drift or auto-correlation).

Effect(s) tested

Define precise effect in terms of the task or stimulus conditions instead of psychological concepts and indicate whether ANOVA or factorial designs were used.

Specify type of analysis: ☐ Whole brain ☐ ROI-based ☐ Both

Statistic type for inference
(See Eklund et al. 2016)

Specify voxel-wise or cluster-wise and report all relevant parameters for cluster-wise methods.

Correction

Describe the type of correction and how it is obtained for multiple comparisons (e.g. FWE, FDR, permutation or Monte Carlo).

Models & analysis

n/a	Involved in the study
☐	☐ Functional and/or effective connectivity
☐	☐ Graph analysis
☐	☐ Multivariate modeling or predictive analysis

Functional and/or effective connectivity

Report the measures of dependence used and the model details (e.g. Pearson correlation, partial correlation, mutual information).

Graph analysis

Report the dependent variable and connectivity measure, specifying weighted graph or binarized graph, subject- or group-level, and the global and/or node summaries used (e.g. clustering coefficient, efficiency, etc.).

Multivariate modeling and predictive analysis

Specify independent variables, features extraction and dimension reduction, model, training and evaluation metrics.
